# Revisiting the Taxonomy of *Cylapocoris* Carvalho, 1954 (Hemiptera: Miridae: Cylapinae) with Descriptions of Five New Species and Morphology-Based Phylogenetic Analysis of the Genus †

**DOI:** 10.3390/insects14090721

**Published:** 2023-08-22

**Authors:** Andrzej Wolski, Adrian Masłowski, Artur Taszakowski

**Affiliations:** 1 Institute of Biology, University of Opole, Oleska 22, 45-052 Opole, Poland; 2 Institute of Biology, Biotechnology and Environmental Protection, Faculty of Natural Sciences, University of Silesia in Katowice, Bankowa 9, 40-007 Katowice, Poland; adrian.maslowski@us.edu.pl

**Keywords:** Heteroptera, Fulviini, systematics, phylogeny, morphology, taxonomy, diagnosis, female genitalia, Neotropics

## Abstract

**Simple Summary:**

*Cylapocoris* is a genus of the cylapine tribe Fulviini distributed in the Neotropics, with most species being recorded from Central America. By the descriptions of five new species and redescriptions of six species, we provide a robust amount of morphological data, including the novel study of female genitalia, offering an updated diagnosis and description of the genus. This paper also elucidates the phylogenetic position of the genus and the interrelationships of the species within it as well as confirming the monophyly of *Cylapocoris*.

**Abstract:**

This paper provides descriptions of five new species of the Neotropical genus *Cylapocoris* Carvalho, 1954 (*C. bimaculatus* n. sp., *C. brooksi* n. sp., *C. carvalhoi* n. sp., *C. scutellatus* n. sp., and *C. simplexoides* n. sp.). *Cylapocoris* and *Cylapocoroides* Carvalho, 1989 are redescribed and rediagnosed. Illustrations of male genitalia, scanning electron micrographs of selected structures of certain taxa, and an identification key to species are provided. Female genitalia are described and illustrated for the first time for *Cylapocoris* in nine out of 19 known species. A cladistic analysis of the genus, based on 62 morphological characters, is presented as a contribution to the understanding of relationships within *Cylapocoris* and its relationships with other groups of Cylapinae. The analysis comprises 16 ingroup species and 15 outgroup taxa. Both equal and implied weighting parsimony analyses were used in the phylogenetic reconstruction. We confirm the monophyly of *Cylapocoris* and its sister-group relationship with *Cylapocoroides*. Additionally, we identify subgroupings within *Cylapocoris*. Intertribal relationships within Cylapinae are briefly discussed.

## 1. Introduction

Cylapinae Kirkaldy, 1903 [1] is a small subfamily within the highly diverse family Miridae Hahn, 1831 (Hemiptera: Heteroptera) currently including over 520 species [2,3,4,5,6,7,8,9]. Most cylapine genera and species are known from the tropics and subtropics. Cylapines are rare in collections owing to cryptic habitats, and many species are represented only by the holotype or a few specimens, with access to molecular data being extremely limited for most taxa. As a result, our understanding of their biology, taxonomy, morphology, and distribution as well as phylogenetic relationships remain poor. Currently, Cylapinae is classified into six tribes, including two large groupings, i.e., Fulviini Uhler, 1886 and Cylapini Kirkaldy, 1903 and four small tribes: Bothriomirini Kirkaldy, 1906, Rhinomirini Gorczyca, 2000, Psallopini Schuh, 1976, and Vanniini Gorczyca, 1997 [10]. However, the identity and placement of those groupings as well as the monophyly of the subfamily remain doubtful [6,9,11]. Our knowledge of the phylogenetic relationships within the family is restricted to a few papers focusing on taxa at the tribal level [4,5,9] or below [12,13,14] with no existing works covering the entire subfamily and most of these studies are based on morphological data, while molecular information is available for only a small number of cylapine taxa.

Although biological data are unavailable for most taxa, association with fungi has been recorded for many species of this group, e.g., [5,15,16,17,18,19].

*Cylapocoris* Carvalho, 1954 is a Neotropical genus originally described by Carvalho [20] to accommodate two species—*C. pilosus* and *C. tiquiensis*. Since then, four additional species have been added by Carvalho and Gomes [21] and Carvalho [22,23,24]. Wolski [25,26] increased the total number of known species to sixteen. The aim of this study is to contribute to the knowledge of *Cylapocoris* by providing descriptions of five new and redescriptions of six known species. Additionally, this study aims to update and consolidate the morphological and taxonomic information available for the genus. Notably, the investigation includes a novel examination of female genitalia. In this study, we also conducted phylogenetic analyses to determine the genus phylogenetic position and the relationships among its constituent species, as well as to assess its monophyly. This study is based solely on morphological characters due to the limited availability of suitable specimens for molecular studies in most taxa.

## 2. Material and Methods

### 2.1. Specimens

This study is based on the material deposited in the following institutions: American Museum of Natural History, New York, NY, USA (AMNH), Natural History Museum, London, United Kingdom (BMNH-NHM), California Academy of Sciences, San Francisco, USA (CAS), Department of Zoology, University of Silesia, Katowice, Poland (DZUS), Institut Royal des Sciences Naturelles de Belgique, Bruseles, Belgium (ISNB); Division of Entomology, Biodiversity Institute Kansas University, Lawrence, USA (KU), División Entomología, Museo Argentino de Ciencias Naturales “Bernardino Rivadavia”, Buenos Aires, Argentina (MACN), Museum National d’Histoire Naturelle, Paris, France (MNHN); Museu Nacional, Rio de Janeiro, Brazil (MNRJ) Naturhistorisches Museum Wien, Vienna, Austria (NHMW), Naturhistoriska Riksmuseet, Stockholm, Sweden (NHRS), National Museum, Prague, Czechia (NMPC), Natural History Department, Tiroler Landesmuseum Innsbruck, Austria (TLM), Tomohide Yasunaga collection (TYCN), Systematic Entomology Laboratory [SEL], ARS, USDA, c/o National Museum of Natural History, Smithsonian Institution, Washington, D.C., USA (USNM), Universidade de São Paulo, Brazil (USP), Zdeněk Jindra collection, Praha, Czech Republic (ZJPC), and Zoologische Staatssammlung, München, Germany (ZSM).

### 2.2. Taxon Selection and Morphological Data

Thirty-one taxa were examined for the analysis. The ingroup comprises 16 representatives of *Cylapocoris*. The outgroup consists of 15 species belonging to other Cylapinae tribes, as defined by Schuh and Weirauch [10]. The tree was rooted with *Isometopus intrusus* (Herrich-Schaeffer, 1835) [27] as the Isometopinae is considered most closely related to Cylapinae [28,29].

A total of 62 morphological characters obtained from the head (25), pronotum (9), scutellum (2), thoracic pleura (5), hemelytron (5), and male (12) and female (4) genitalia were coded. Forty-six characters are binary, and 21 are multistate. Missing data were coded as “?” and inapplicable characters were coded as “–“. All characters were treated as unordered, which means that any character state can change to any other character state at the cost of a single step [30] and were initially equally weighed [31], thus making no assumptions about character evolution. The morphological data were obtained from personal observations and from referencing states cited in previous phylogenetic studies [4,5,7,8,11]. The dataset was managed and edited in Mesquite v3.61 software [32]. The matrix is given in the Supplementary File S1.

Habitus photographs were made in the Laboratory of Insect Anatomy and Morphology, Institute of Biology, Biotechnology and Environmental Protection, the University of Silesia in Katowice (Katowice, Poland) as follows: the focus-stacked, color photographs were prepared with a Leica M205C stereo microscope with a high diffuse dome illumination Leica LED5000 HDI, Leica DFC495 digital camera, and Leica application suite 4.12.0 software (Leica Microsystems, Vienna, Austria).

SEM micrographs were prepared in the Educational Laboratory of Scanning Microscopy, Institute of Biology, Biotechnology and Environmental Protection, the University of Silesia in Katowice (Katowice, Poland) using Phenom XL scanning electron microscope (Phenom-World B.V., Eindhoven, The Netherlands) at 15 kV accelerating voltage with a BackScatter Detector (BSD). Specimens were uncoated, only cleaned with a micro brush.

The males’ genitalia structures were imaged in the Laboratory of Insect Anatomy and Morphology, Institute of Biology, Biotechnology and Environmental Protection, the University of Silesia in Katowice (Katowice, Poland) as follows: using a Leica DM 3000 upright light microscope with Leica MC 190 HD digital camera and Leica Application Suite 4.12.0 software. Male genitalia and female abdomen were separated from the insect body using standard entomological pins and a hypodermic needle (0.45 × 16 mm). Then, they were macerated in a 5% solution of KOH for 24 h (room temperature) and viewed with a stereoscopic microscope to separate the aedeagus from the genital capsule and remove parts of the cuticle that obscure the view [33].

Images of the female genitalia were taken in the Laboratory of Insect Systematics and Ecology, Institute of Biology, University of Opole (Opole, Poland) using Olympus BX upright light microscope with Canon EOS 750D digital camera. Multiple image layers were stacked using Helicon Focus v7.6.1 software. The female abdomen was macerated in a 10% solution of KOH for 24 h (room temperature) and stained with chlorazol black [34].

To obtain high-quality figures, fragments of specimens (for both light microscopy and SEM) were imaged at high magnifications. Then, photographs were combined using the Image Composite Editor (panoramic image stitcher). The figures were prepared using Adobe Photoshop CS6 graphic editor.

Measurements were made using an eyepiece (ocular) micrometer and are presented in millimeters (mm).

Adult terminology used in the text follows Schuh and Weirauch [10]. The measured body parts were defined by Wolski [35]. Terminology of female genitalia follows [10,36,37]. Terminology of the male genitalia follows [38,39]. Terms for the endosomal elements of *Cylapocoris* proposed by Wolski [25] are abbreviated as follows:

AS—apical sclerite—rounded, strongly sclerotized lobe situated apically on the endosoma;
BS—basal sclerite—sclerite situated basally on the endosoma;
DSS—sclerotized portion of the ductus inside the endosoma;
LS—lateral sclerite—situated laterally on the endosoma;
ML—medial lobe—sclerotized, serrate lobe, sometimes occupying most of endosoma, usually situated medially;
MS—mesial sclerite—situated medially on the endosoma.

### 2.3. Phylogenetic Analysis

The dataset was analyzed under parsimony with TNT v1.5 (traditional search, 10,000 replicates, 100 trees saved per replicate) [40]. Heuristic searches were performed under both equal (EW) and implied (IW) weighting [40,41]. The latter was aimed to minimize the effect of potentially homoplastic characters over phylogenetic signal, down-weighting them to different extents, depending on the selected penalty factor (concavity constant, *k*) [41]. In this study, *k*-values were set at 1–10 (from strong to weaker down-weight). Character states were mapped on a maximum parsimonious tree using Winclada [42], showing only unambiguous changes. All unsupported nodes were collapsed after each analysis.

**Figure 1:**
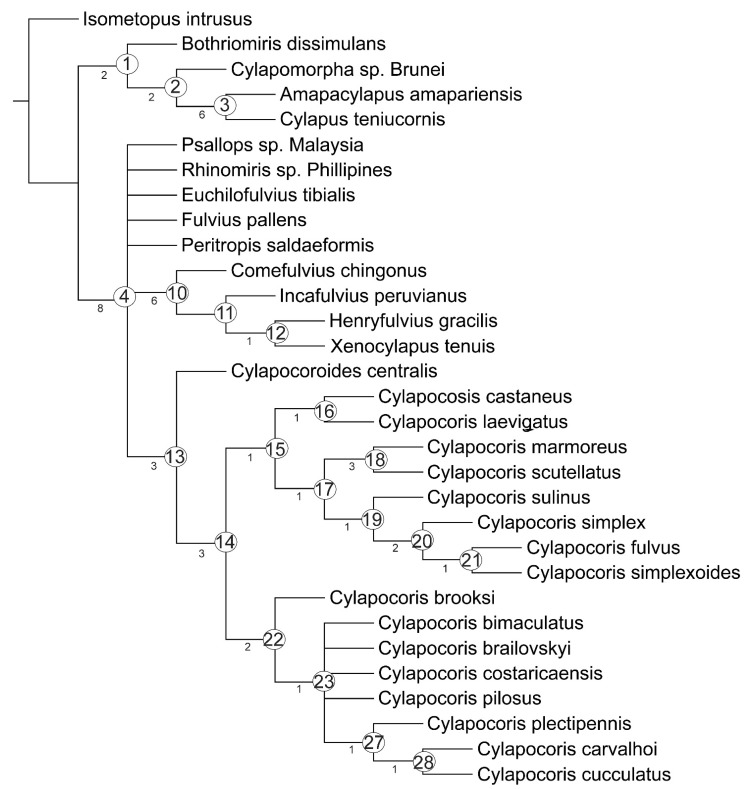
Strict consensus tree obtained from 6 most parsimonious trees under equal weights. Bremer support values are indicated below branches.

**Figure 2:**
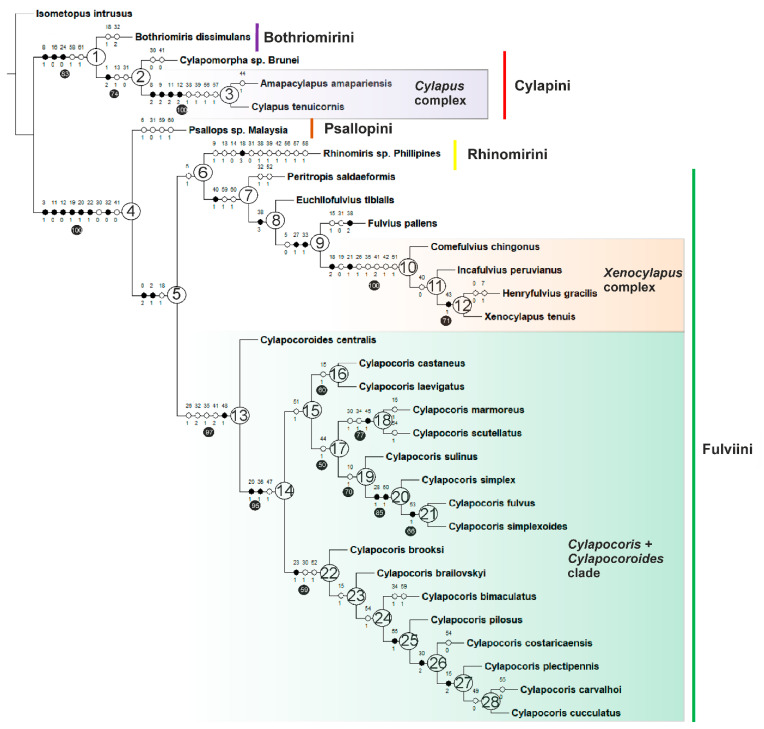
Strict consensus tree obtained from 2 most parsimonious trees using implied weighting of characters (k = 3). Numbers of nodes are in circles. Non-homoplastic changes in character states are represented with black circles, homoplastic changes with white circles. Symmetric resampling supports (SRS) are indicated below branches.

Bremer support values (BS) [43] were obtained in TNT for the unweighted analysis (Figure 1). Symmetric resampling supports (SRS) [44] were also performed for the implied weights analysis in TNT. Unlike bootstrap and jackknife values, symmetric resampling values are not affected by character weight and transformation costs [40,44]. Statistics > 50% are given in Figure 2 within closed circles.

#### List of Characters (Figure 3, Figure 4, Figure 5, Figure 6, Figure 7, Figure 8, Figure 9, Figure 10, Figure 11, Figure 12, Figure 13, Figure 14, Figure 15, Figure 16, Figure 17, Figure 18, Figure 19, Figure 20 and Figure 21)

**Head structure**

**Figure 3:**
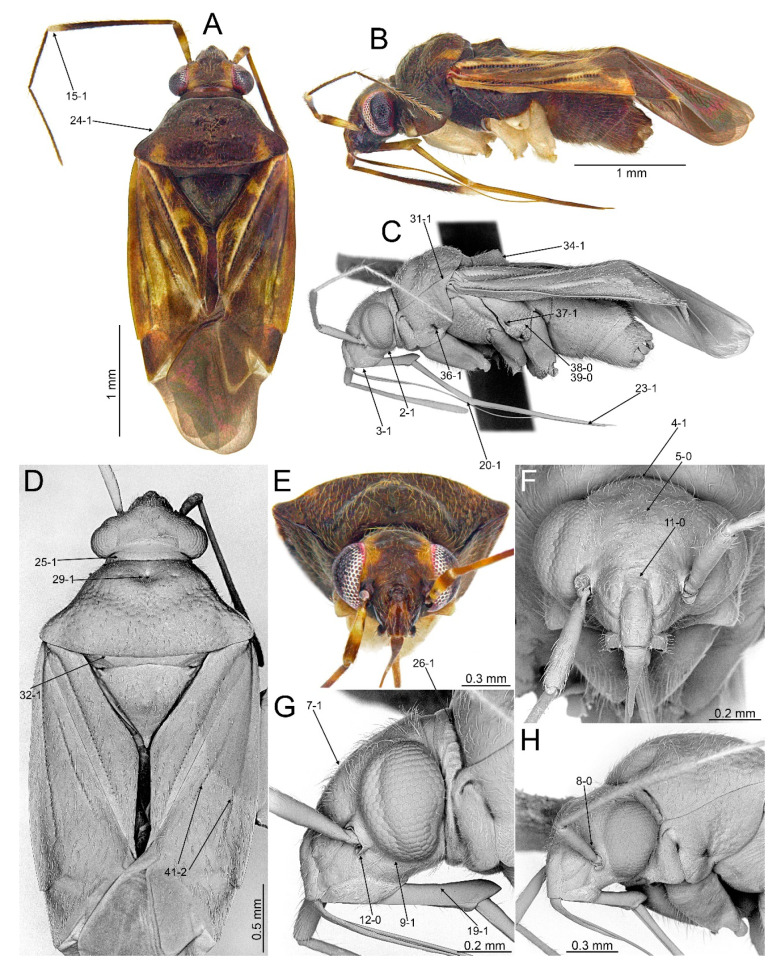
*Cylapocoris bimaculatus* sp. nov., holotype: (**A**,**D**). Dorsal view; (**B**,**C**). Lateral view; (**E**). Head and pronotum, anterior view; (**F**). Head, anterior view; (**G**). Head, lateral view; (**H**). Head and pronotum, anterolateral view.

**Figure 4:**
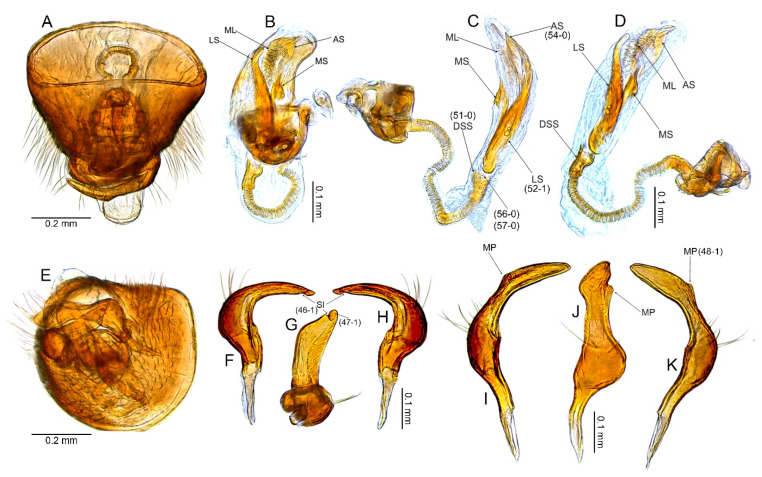
*Cylapocoris bimaculatus* sp. nov., male genitalia, holotype: (**A**,**E**). Pygophore: (**A**). dorsal view, (**E**). Caudal view; (**B**). Aedeagus with theca, dorsal view; (**C**,**D**). Aedeagus, theca removed: (**C**). Left lateral view, (**D**). Right lateral view; (**F**–**H**). Left paramere: (**F**). Right lateral view, (**G**). Apical process, ventral view; (**H**). Left lateral view; (**I**–**K**). Right paramere: (**I**). Right lateral view; (**J**). Apical process, dorsal view, (**K**). Left lateral view. AS = apical sclerite; DSS = sclerotized part of ductus seminis inside endosoma; LS = lateral sclerite; ML = mesial lobe; MP = medial process; MS = mesial sclerite; SI = subapical incision.

**Figure 5:**
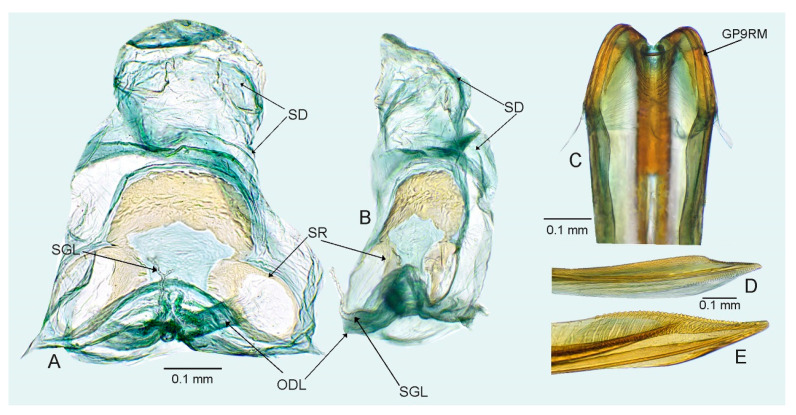
*Cylapocoris bimaculatus* sp. nov., female genitalia, paratype: (**A**,**B**). bursa copulatrix: (**A**). Dorsal view, (**B**). Anterolateral view; (**C**). Posterior wall of bursa copulatrix; (**D**,**E**). Ovipositor: (**D**). Gonapophysis 8, (**E**). Gonapophysis 9. GP9RM = ramus of gonapophysis 9; ODL = lateral oviducts; SD = seminal depository; SGL = spermathecal gland; SR = sclerotized rings.

**Figure 6:**
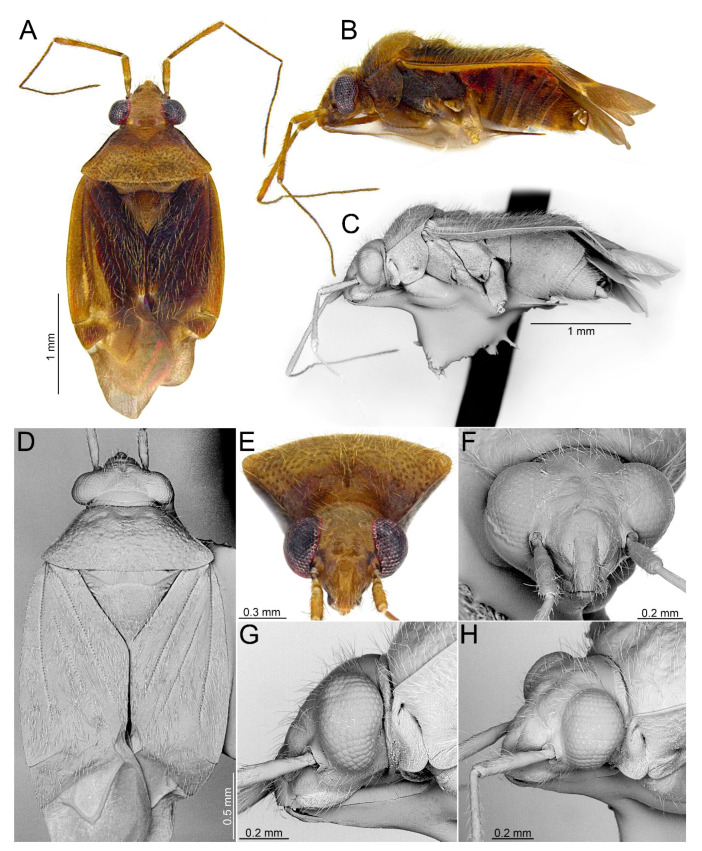
*Cylapocoris brooksi* sp. nov., holotype: (**A**,**D**). Dorsal view; (**B**,**C**). Lateral view; (**E**). Head and pronotum, anterior view; (**F**). Head, anterior view; (**G**). Head, lateral view; (**H).** Head and pronotum, anterolateral view.

**Figure 7:**
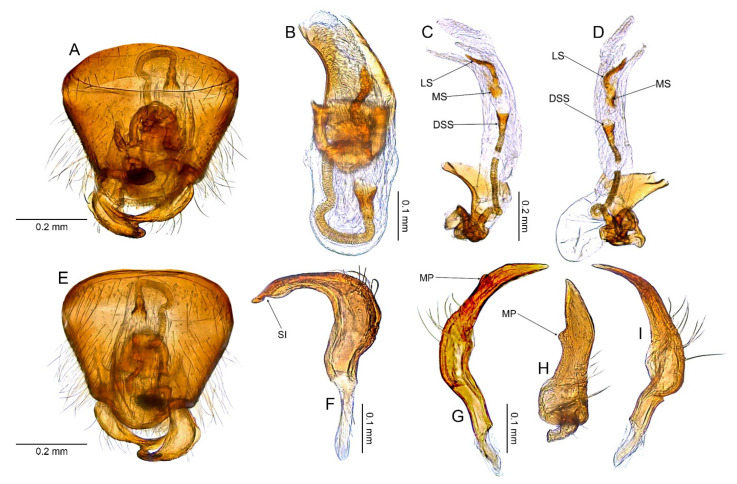
*Cylapocoris brooksi* sp. nov., male genitalia, holotype: (**A**,**E**). Pygophore: (**A**). Dorsal view, €. Ventral view; (**B**). Aedeagus with theca, dorsal view; (**C**,**D**). Aedeagus, theca removed: (**C**). Left lateral view, (**D**). Right lateral view; (**F**). Left paramere, left lateral view; (**G**–**I**). Right paramere: (**G**). Right lateral view; (**H**). Apical process, ventral view, **I**. Left lateral view. DSS = sclerotized part of ductus seminis inside endosoma; LS = lateral sclerite; MP = medial process; MS = mesial sclerite; SI = subapical incision.

0. Head orientation: hypognathous (e.g., Figure 2C in [6]; Figure 9a–d in [9]) (0); porrect (e.g., Figure 3A–C and Figure 6A–C) (2).
1. Head shape: not flattened dorsoventrally (e.g., Figure 3A–C and Figure 6A–C) (0); moderately flattened dorsoventrally (1); strongly flattened dorsoventrally (e.g., Figure 9 in [26]) (2).
2. Gula development: shorter than diameter of eye (0) (e.g., Figure 9 in [26], Figure 3i in [6]); as long as or longer than diameter of eye (e.g., Figure 3B,C and Figure 6B,C) (1).
3. Buccula shape: rounded (e.g., Figures 5C and 8C in [4]; Figure 9a–d in [9]) (0); elongated (e.g., Figures 3B,C and 9a–f in [9]) (1).
4. Vertex posterior carina: absent (e.g., Figure 9e in [9]) (0); present (e.g., Figure 3D,F and Figure 6D) (1).
5. Longitudinal sulcus on vertex presence: absent (e.g., Figure 3F) (0); present (e.g., Figure 25 in [45]) (1).
6. Width/height ratio of head, anterior view: about twice higher than wide (e.g., Figure 8d in [9]) (0); as high as wide or slightly wider than high (e.g., Figure 8i in [9]) (1); about twice wider than high (e.g., Figure 3F) (2).
7. Position of frons in relation to vertex: located at right or acute angle to vertex (e.g., Figure 9 in [26]) (0); sloping (e.g., Figure 9d in [9]) (1); forming obtuse angle with vertex (e.g., Figure 3B,C,G) (2).
8. Posterior suture of mandibular plate presence and development: absent (e.g., Figure 3G,H and Figure 6G; Figure 9e in [9]) (0); shallow, weakly developed (e.g., Figure 9d in [9]) (1); deep, strongly developed (e.g., Figure 9 in [26]) (2).
9. Eye position in lateral view: ventral margin of eye reaching ventral margin of head (e.g., Figure 3F and Figure 6G; Figure 9e in [9]) (0); ventral margin of eye slightly removed from ventral margin of head (Figure 9d in [9]) (1); ventral margin of eye strongly removed from ventral margin of head (e.g., Figures 9 and 123 in [26]) (2).
10. Portion between apex of clypeus and anterior margin of eye length: longer than eye width in lateral view (e.g., Figure 3G and Figure 6G) (0); shorter than eye width in lateral view (e.g., Figure 17C) (1).
11. Position of clypeal base: distinctly above ventral margin of eye (e.g., Figure 3F and Figure 6F; Figure 8l in [9]) (0); slightly below ventral margin of eye (Figure 8i in [9]) (1); distinctly below ventral margin of eye (Figure 9 in [26]) (2).
12. Antennal insertion position: bordering suture between maxillary and mandibular plates (e.g., Figure 3G and Figure 6G; Figure 13I in [7]) (0); slightly removed from suture between maxillary and mandibular plates in dorsal direction (as in Figure 9b,d in [9]) (1); strongly removed from suture between maxillary and mandibular plates in dorsal direction (as in Figure 9a,c in [9]) (2).
13. Total antennal length: length equal to median of body or weakly beyond (e.g., Figure 3A and Figure 6A) (0); much longer than body length (as in Figures 4b,c, 7d and 11n in [9]) (1).
14. Antennomere I length: significantly longer than vertex width (0); as long as or slightly longer than vertex width (1).
15. Pale annulation on antennomere II apically presence: absent (Figure 6A, Figure 15A and Figure 17A) (0); present (Figure 3A, Figure 9A and Figure 12D) (1).
16. Labium structure: thin, sharply pointed (e.g., Figure 3C) (0); stout (e.g., Figure 9j,k in [9]) (1).
17. Labium length: short, reaching hind coxae (Figure 9j,k in [9]) (0); reaching well beyond hind coxae (e.g., Figure 3C and Figure 9C) (1).
18. Labial segment I length: more than two times longer than gula length (Figure 9 in [9]) (0); as long as gula length or moderately longer (e.g., Figure 3C) (1); about a half of gula length (Figure 8 in [46], Figures 34 and 35 in [35]) (2); about two times longer than gula length (3).
19. Structure of labial segment I: not subdivided (as in Figure 9c,j,k in [9]; Figure 66 in [35]) (0); subdivided (e.g., Figure 3C,G and Figure 6C) (1).
20. Structure of labial segment II: not subdivided (as in Figure 9i,k in [9]) (0); subdivided (e.g., Figure 3C and Figure 13A) (1).
21. Ladder-like structure on lateral surface of labial segment II presence: absent (0); present (Figure 66 in [35], Figure 5 in [47]) (1).
22. Labial segments I/IV length ratio: segment IV about two times shorter than segment I (Figure 9j,k in [9]) (0); segment IV subequal to segment I (e.g., Figure 3C and Figure 6C) (1).
23. Labial segment IV structure: weakly pointed (Figure 9j,k in [9]) (0); sharply pointed (e.g., Figure 3C and Figure 6C) (1).

**Pronotum and scutellum**

24. Lateral carina of pronotum in dorsal view: not visible or weakly visible (Figure 17A) (0); well visible (e.g., Figure 3A,D and Figure 6A,D) (1).
25. Pronotal collar structure in dorsal view: depressed (e.g., Figure 3G and Figure 6G) (0); convex (e.g, Figures 66 and 67 in [35]) (1).
26. Suture delimiting pronotal collar: smooth (0); scalloped (e.g., Figure 3G and Figure 6G) (1).
27. Pronotal calli length: shorter than posterior lobe of pronotum (e.g., Figure 3A and Figure 6A) (0); longer than posterior lobe of pronotum (e.g., Figure 67 in [35]) (1).
28. Pronotal length: about two times longer than wide (e.g., Figure 3A and Figure 6A) (0); less than two times longer than wide (Figure 17A; Figure 4 in [25]) (1).
29. Pit between calli presence: absent (0); present (e.g, Figure 3D and Figure 6D) (1).
30. Punctation on pronotum presence: absent or weakly developed (e.g., Figure 17A,E; Figures 4 and 38 in [25]) (0); moderately developed (e.g., Figure 3A,D and Figure 6A,D) (1); strongly developed (e.g., Figure 9A,D; Figure 37 in [25]) (2).
31. Lateral margin of pronotum structure: not carinate (e.g., Figure 9e in [9]) (0); carinate (e.g., Figure 3C,H and Figure 6C,H) (1).
32. Posterior margin of pronotum shape: concave (e.g., Figure 2 in [46]; Figure 36 in [35]) (0); sinuate or weakly concave (e.g., Figure 33 in [9]) (1); straight (e.g., Figure 3A and Figure 6A; Figure 1 in [48]) (2).
33. Humeral angle shape: short, broad (e.g., Figure 3A and Figure 6A) (0); long, narrow (e.g., Figure 2 in [46]; Figure 36 in [35]) (1).
34. Scutellum structure: flat or weakly arched (e.g., Figure 3B,C and Figure 9B) (0); with more or less developed swelling medially (e.g., Figure 15B) (1).
35. Lateral margin of scutellum structure: smooth (0); scalloped (Figure 39 in [25]) (1).

**Thoracic pleura**

36. Deep apodeme on proepimeron: absent (0); present (e.g., Figure 3C and Figure 6C) (1).
37. Apodeme on anteroventral portion of metepisternum: absent (0); present (e.g., Figure 3C and Figure 6C) (1).
38. Scent gland evaporative area development: occupying ventral margin of metepisternum (e.g., Figure 3C and Figure 6C) (0); well expanded onto anterior margin of metepisternum (e.g., Figure 10b,c in [9]; Figure 22A in [5]) (1); restricted to posterior portion of metepisternum (as in Figure 31 in [49]) (2); absent (e.g., Figure 32 in [45]; Figure 68 in [35]) (3).
39. Scent gland evaporative area shape: more or less triangular (e.g., Figure 3C and Figure 6C) (0); semicircular (e.g., Figure 10b,c in [9]; Figure 22A in [5]) (1); ovoid (as in Figure 31 in [49]) (2).

**Hemelytron**

**Figure 8:**
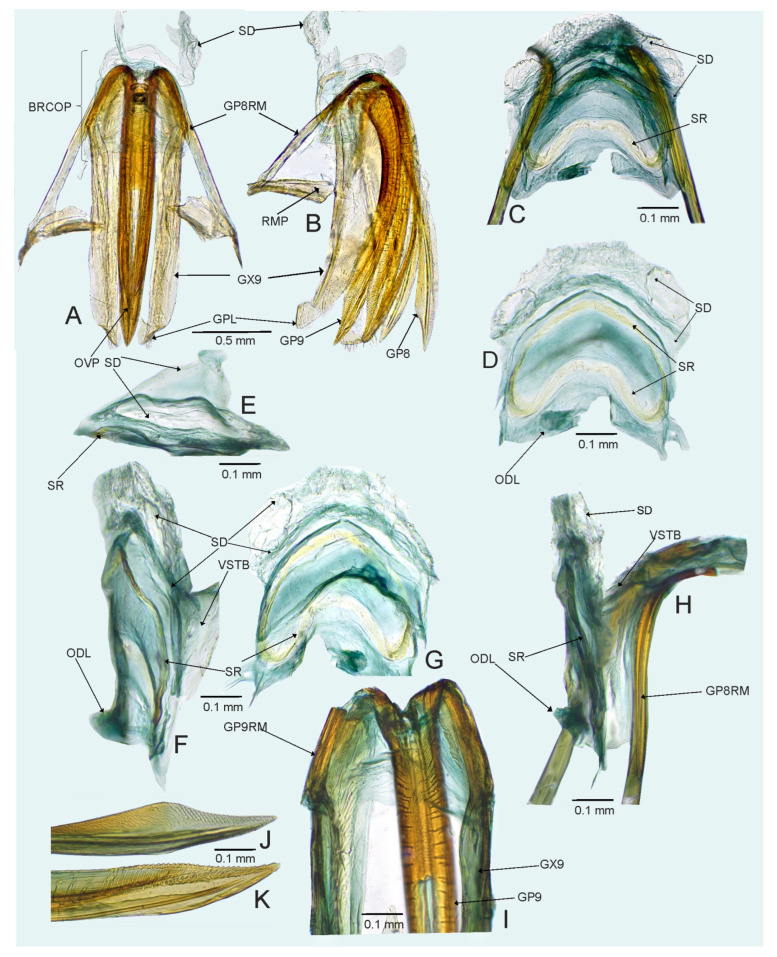
*Cylapocoris brooksi* sp. nov., paratype: (**A**,**B**). Female genitalia: (**A**). Dorsal view, (**B**). Lateral view; (**C**,**D**). Bursa copulatrix, dorsal view; (**E**). Bursa copulatrix, anterior view; (**F**,**H**). Bursal copulatrix, lateral view; (**G**). Bursa copulatrix, ventral view; **I**. Bursa copulatrix, posterior wall; (**J**,**K**). Ovipositor: (**J**). Gonapophysis 8, (**K**). Gonapophysis 9. BRCOP = bursa copulatrix; GP8 = gonapophysis 8; GP9 = gonapophysis 9; GP8RM = ramus of gonapophysis 8; GP9RM = ramus of gonapophysis 9; GPL = gonoplac; GX9 = gonocoxae 9; ODL = lateral oviducts; OVP = ovipositor; RMP = ramal plate; SD = seminal depository; SGL = spermathecal gland; SR = sclerotized rings; VSTB = vestibulum.

**Figure 9:**
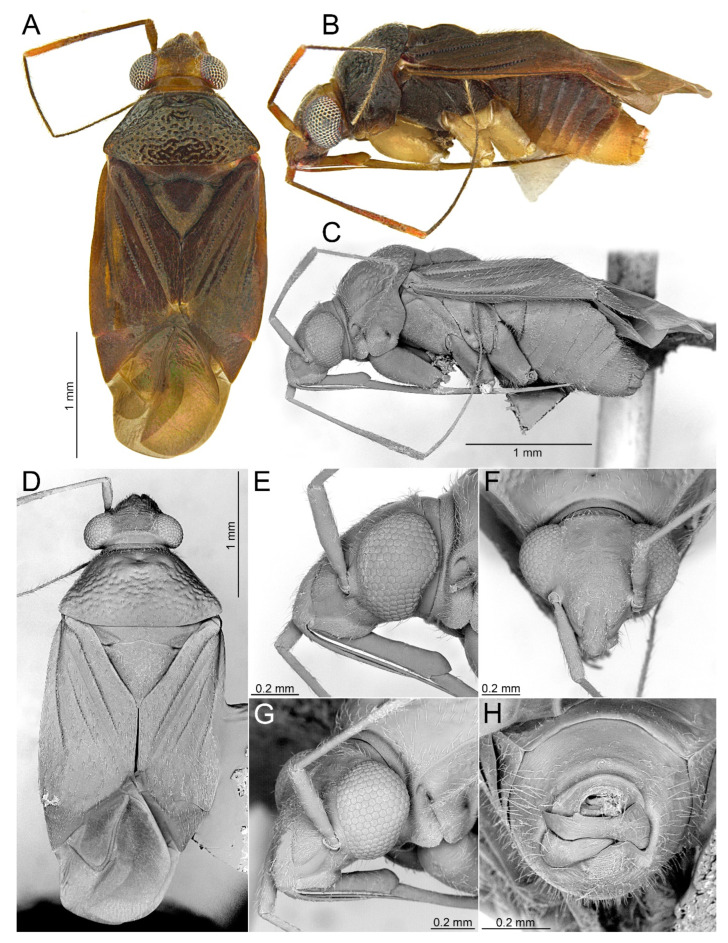
*Cylapocoris carvalhoi*, holotype: (**A**,**D**). Dorsal view; (**B**,**C**). Lateral view; (**E**). Head, lateral view; (**F**). Head, anterior view; (**G**). Head and pronotum, anterolateral view; (**H**). Pygophore, caudal view.

40. Setae on hemelytron: simple (e.g., Figure 3A–D and Figure 6A–D) (0); scalelike (e.g., Figure 9e in [9]) (1).
41. Punctation on hemelytron: absent (e.g., Figure 33 in [26]; Figures 42 and 43 in [49]) (0); present, uniform (Figures 8 and 120 in [26]) (1); hemelytron with at least one row of punctures (e.g., Figure 3D and Figure 6D) (2).
42. Costal fracture presence: present (0); absent (Figures 1 and 2 in [46]; Figure 71 in [35]) (1).
43. Brown yellow regular pattern between medial fracture and R + M vein: absent (0); present (Figures 1–14 in [35]) (1).
44. Yellowish patch on apex of endocorium presence: absent (0); present (Figure 15A and Figure 17A; Figures 4 and 9–11 in [25]) (1).
45. Yellow, regular, relatively large patches on hemelytron presence: absent (0); present (Figure 15A; Figure 8 in [25]) (1).

**Male genitalia**

**Figure 10:**
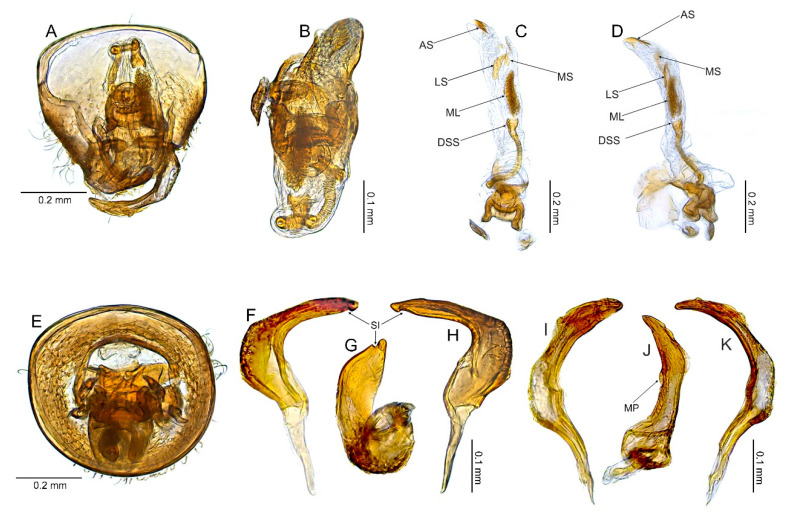
*Cylapocoris carvalhoi* sp. nov., male genitalia, holotype: (**A**,**E**). Pygophore: (**A**). dorsal view, (**E**). Caudal view; (**B**). Aedeagus with theca, dorsal view; (**C**,**D**). Aedeagus, theca removed: (**C**). Ventral view, (**D**). Left lateral view; (**F**–**H**). Left paramere: (**F**). Right lateral view, (**G**). Apical process, ventral view, (**H**). Left lateral view; (**I**–**K**). Right paramere: (**I**). Right lateral view, (**J**). Apical process, ventral view, (**K**). Left lateral view. AS = apical sclerite; DSS = sclerotized part of ductus seminis inside endosoma; LS = lateral sclerite; ML = mesial lobe; MP = medial process; MS = mesial sclerite; SI = subapical incision.

46. Left paramere apical process subapical incision presence: absent (0); present (e.g., Figure 4F–H and Figure 10F–H; Figures 27 and 32 in [25]) (1).
47. Left paramere apical process in dorsal view: pointed or straight (0); rounded (e.g., Figure 4G and Figure 10G; Figures 27 and 32 in [25]) (1).
48. Right paramere apical process medial outgrowth presence: absent (0); present (e.g., Figure 4I–K and Figure 7G,H) (1).
49. Ratio between lateral sclerite (LS)/mesal sclerite (MS): LS about as large as MS (e.g., Figure 10C,D; Figure 40 in [25]) (0); LS much larger than MS (e.g., Figure 4C,D) (1).
50. Endosoma thickness: inflated (e.g., Figure 4C,D and Figure 7C,D) (0); thin (Figure 16D and Figure 18D) (1).
51. Sclerotized part of ductus seminis inside endosoma length: longer than wide (e.g., Figure 4C,D and Figure 7C,D) (0); as long as wide (Figure 16D and Figure 18D) (1).
52. Lateral sclerite presence: absent (e.g., Figure 16D and Figure 18D) (0); present (e.g., Figure 4C,D and Figure 7C,D) (1).
53. Basal sclerite presence: absent (e.g., Figure 4C,D and Figure 7C,D) (0); present (Figure 18D; Figure 45 in [25]) (1).
54. Apical sclerite presence: present (e.g., Figure 4C,D and Figure 10C,D) (0); absent (e.g., Figure 7C and Figure 18D) (1).
55. Apical sclerite development: thin (e.g., Figure 4B–D) (0); broadened (Figure 10C,D; Figure 62 in [25]) (1).
56. Secondary gonopore development: undifferentiated or weakly developed (e.g., Figure 4C and Figure 7C) (0); well developed (Figures 12 and 135 in [26]) (1).
57. Microsculpture around secondary gonopore: absent (e.g., Figure 4C and Figure 7C) (0); present (e.g., Figures 12 and 135 in [26]) (1).

**Female genitalia**

**Figure 11:**
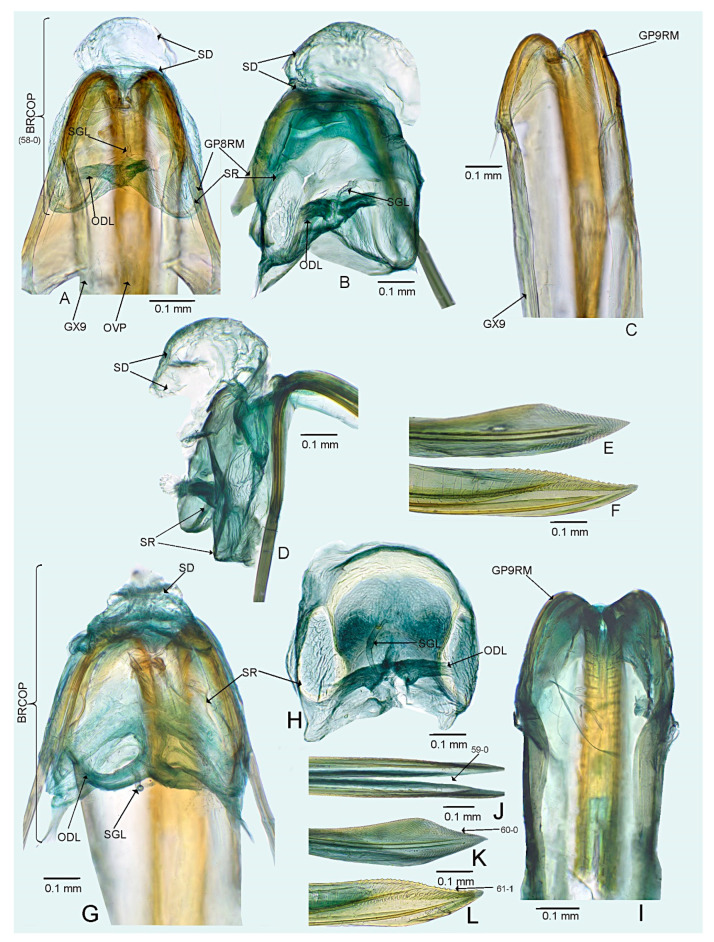
*Cylapocoris cucculatus* Wolski, 2013, female from Costa Rica (**A**–**F**). and *C. funebris* (Distant, 1883), female from Costa Rica (**G**–**I**): (**A**,**B**,**D**,**G**,**H**). Bursa copulatrix: (**A**,**B**,**G**,**H**). Dorsal view, (**D**). Lateral view; (**C**,**I**). Bursa copulatrix, posterior wall; (**E**,**F**,**J**–**L**): Ovipositor: (**J**). Gonapophyses 8; (**E**,**K**). Gonapophysis 8, (**F**,**L**). Gonapophysis 9. BRCOP = bursa copulatrix; GP8RM = ramus of gonapophysis 8; GP9RM = ramus of gonapophysis 9; GX9 = gonocoxae 9; ODL = lateral oviducts; OVP = ovipositor; SD = seminal depository; SGL = spermathecal gland; SR = sclerotized rings.

58. Bursa copulatrix size: thin, not extending beyond first gonapophyses (e.g., Figure 11A,B) (0); thick, extending beyond gonapophyses 8 (e.g., Figures 7F and 10F in [50]; Figure 17i in [9]) (1).
59. Membranous structure between eight gonapophyses presence: absent (e.g., Figure 11J), (0); present (as in Figure 5 in [51]) (1).
60. Gonapophyses 8 apex shape: tapering toward apex, pointed (e.g., Figure 5D and Figure 11K) (0); obtuse (Figure 5C in [50]) (1).
61. Gonapophyses 9 teeth presence: absent (0); present (Figure 5E, Figure 8K and Figure 11L; Figure 21j,l in [9]) (1).

### 2.4. Map

The map was prepared in SAGA GIS 7.1.1 (http://www.saga-gis.org (accessed on 12 March 2023)) using WGS84 datum and EPSG: 3395 (World Mercator cylindrical projection) [52].

## 3. Results

### 3.1. Phylogeny

The heuristic search with equal character weights of characters produced equally the six most parsimonious trees of 153 steps, with a consistency index CI = 53 and a retention index RI = 77. The strict consensus tree with Bremer support values for each branch is shown in Figure 1.

Each of the *k* = 3–10 IW analyses resulted in two trees. The tree topologies differed in terms of position of several ingroup taxa, but the major clades remained unaffected. Character optimizations are given for a consensus tree obtained from the implied weighting analysis with *k*-value = 3 (Figure 2), which had the highest weighting impact.

The phylogenetic analyses using the EW and IW approaches yielded similar results in terms of establishing the phylogenetic relationships within the subfamily Cylapinae. The analyses identified two main clades within the subfamily: clade 1 which consists of Bothriomirini and Cylapini, and it received high nodal support in both analyses (BS 2, SRS 83%) and clade 4 which comprises Fulviini, Psallopini, and Rhinomirini. It also received high nodal support in both analyses (BS 8, SRS 100%). The relationships within clade 1 were consistent between the EW and IW topologies. The analyses revealed a sister-group relationship of Bothriomirini to Cylapini, a moderately supported clade (clade 2) containing taxa of Cylapini (BS 2, SRS 74%), and a decisively supported lineage (BS 6, SRS 100%) including taxa of the *Cylapus* complex *sensu* Wolski [9].

Within the clade 4, the relationships were not fully resolved. *Psallops* Usinger, 1946, *Rhinomiris* Kirkaldy, 1902, *Euchilofulvius tibialis* Poppius, 1909, *Fulvius pallens* Gorczyca, 2002, and *Peritropis saldaeformis* Uhler, 1891 formed an unresolved polytomy with the remaining taxa in the EW analysis. However, the IW analysis recovered more resolved relationships compared to the EW analysis, although most of the clades lacked support or had only moderate support. There was a well-supported assemblage (BS 6, SRS 100%) composed of *Comefulvius chingonus* Carvalho and Carpintero, 1985, *Incafulvius peruvianus* Carvalho, 1976, *Henryfulvius gracilis* Wolski, 2015, and *Xenocylapus tenuis* Wolski, 2015 (clade 10, *Xenocylapus* complex) in both the EW and IW analyses. Within this grouping, the analysis showed a sister-group relationship of *Incafulvius peruvianus* to *Henryfulvius gracilis* + *Xenocylapus tenuis* (clade 11), but this relationship lacked statistical support. The latter lineage (*Henryfulvius gracilis* + *Xenocylapus tenuis*) was moderately supported in IW analysis (SRS 73%). Both the EW and IW analyses recovered a sister-group relationship within a well-supported assemblage (BS 3, SRS 97%) containing *Cylapocoroides* and *Cylapocoris* (clade 13). Additionally, the monophyly of *Cylapocoris* was well supported (BS 3, SRS 95%). Within *Cylapocoris*, both the EW and IW analyses identified two subgroupings: *simplex* clade (node 15) and *pilosus* clade (node 22). In the IW analysis, the topology for *Cylapocoris* was resolved, but many of the subordinate clades within the genus lacked support or had weak to moderate support while the EW analysis revealed an unresolved polytomy of *Cylapocoris bimaculatus*, *C*. *braylovskyi*, *C*. *costaricaensis*, and *C. pilosus* with other taxa within the clade 23.

Character optimization

**Node 1**. Bothriomirini + Cylapini. This clade is supported by 83% SRS and the following synapomorphies: posterior suture of mandibular plate shallow, weakly developed (8-1), labium stout (16-1), and labial segment IV weakly pointed (23-0). It is also supported by two homoplasious characters: bursa copulatrix thick, extending beyond gonapophyses 8 (58-1) and gonapophyses 9 teeth present (61-1).

**Node 2**. Cylapini is reconstructed as monophyletic by 74% SRS, one synapomorphy: head strongly flattened dorsoventrally (1-2), and two homoplasious characters: antenna much longer than body length (13-1), lateral margin of pronotum structure not carinate (31-0).

**Node 3**. *Cylapus* complex (*sensu* Wolski 2021 [9]). This group is rendered as monophyletic with high (100%) statistical support and four synapomorphies: posterior suture of mandibular plate deep, strongly developed (8-2), ventral margin of eye strongly removed from ventral margin of head (9-2), clypeal base situated distinctly below ventral margin of eye (11-2), antennal insertion strongly removed from suture between maxillary and mandibular plates in dorsal direction (12-2). This clade is also supported by four homoplasious features: scent gland evaporative area well expanded onto anterior margin of metepisternum (38-1), scent gland evaporative area semicircular (39-2), secondary gonopore well developed (56-1), and microsculpture around secondary gonopore present (57-1).

**Node 4.** The lineage comprising Psallopini + Rhinomirini + Fulviini is statistically well supported with 100% SRS and is supported by seven synapomorphies: buccula elongate (3-1), clypeal base situated distinctly above ventral margin of eye (11-0), antennal insertions bordering suture between maxillary and mandibular plates (12-0), labial segment I subdivided (19-1), labial segment II subdivided (20-1), labial segment IV subequal in length to segment I (22-1); posterior margin of pronotum concave (32-0). This node is also supported by two homoplasious features: punctation on pronotum absent (30-0) and punctation on hemelytron absent (41-0).

**Node 5**. Fulviini + Rhinomirini. This clade is phylogenetically defined by two synapomorphies: head porrect (0-1) and gula as long as or longer than diameter of eye (2-1) and one homoplasious character: labial segment I as long as gula length or moderately longer (18-1). The statistical support for this clade was insignificant.

**Node 6**. Rhinomirini + Fulviini (less *Cylapocoroides* and *Cylapocoris*). This clade is supported by a single homoplasious character: longitudinal sulcus on vertex presence: present (5-1). The statistical support for this clade was insignificant.

**Node 7**. Fulviini (less *Cylapocoroides* and *Cylapocoris*). This node is supported by one synapomorphy: setae on hemelytron scalelike (40-1) and two homoplasious characters: membranous structure between first gonapophyses present (59-1) and first valvula apex obtuse (60-1). The statistical support for this lineage is insignificant.

**Node 8**. This clade is supported by a single synapomorphy: scent gland evaporative area absent (38-3). The support values are insignificant.

**Node 9**. *Fulvius* + *Xenocylapus* complex. This group’s synapomorphies are: pronotal calli longer than posterior lobe of pronotum (27-1) and humeral angle long, narrow (33-1) and one homoplasious character: longitudinal sulcus on vertex absent (5-0). The statistical support for this assemblage is insignificant.

**Node 10**. *Xenocylapus* complex. This assemblage is recovered as monophyletic by a high support value (100% SRS) and contains two synapomorphies: labial segment I about a half of gula length (18-2) and ladder-like structure on lateral surface of labial segment II present (21-1). It is also delimited by following homoplasies: labial segment I not subdivided (19-0), suture delimiting pronotal collar scalloped (26-1); lateral margin of scutellum scalloped (35-1); hemelytron with at least one row of punctures (41-2); costal fracture absent (as in Figure 78 Wolski 2013 [25]) (42-1), sclerotized part of ductus seminis inside endosoma as long as wide (51-1).

**Node 11**. *Incafulvius + Henryfulvius + Xenocylapus*. This node is supported by one homoplasious feature: setae on hemelytron simple (40-0). The support values are insignificant.

**Node 12**. *Henryfulvius + Xenocylapus.* This clade is supported with 71% SRS value and one synapomorphy: hemelytron with brown yellow regular pattern between medial fracture and R + M vein (43-1).

**Node 13**. The monophyly of the clade composed of *Cylapocoroides Cylapocoris* is strongly supported by a high (97%) SRS value and one synapomorphy: right paramere apical process medial process outgrowth (48-1) and four homoplasious traits: suture delimiting pronotal collar scalloped (26-1), posterior margin of pronotum straight (32-2), lateral margin of scutellum structure scalloped (35-1) and hemelytron with at least one row of punctures (e.g., Figure 3D and Figure 6D) (41-2).

**Node 14**. *Cylapocoris*. This group is decisively supported with 95% SRS and two synapomorphies: pit between calli present (29-1), deep cavity on proepimeron present (36-1), and one homoplasy: left paramere apical process rounded in dorsal view (47-1).

**Node 15**. *Cylapocoris simplex* clade. This lineage is phylogenetically defined by one homoplasious character: sclerotized part of ductus seminis inside endosoma as long as wide (51-1). The support values are insignificant.

**Node 16**. *C. castaneus* + *C. laevigatus*. This lineage is monophyletic with 60% SRS and is defined by one homoplasious character: pale annulation on antennomere II apically present (15-1).

**Node 17**. This clade is supported by one homoplasious feature. Yellowish patch on apex of endocorium present (44-1). This clade is recovered as monophyletic by 50% SRS.

**Node 18**. *C. marmoreus + C. scutellatus*. This clade is monophyletic with 77% SRS and is defined by two homoplasious characters: punctation on pronotum present, moderately developed (30-1) and scutellum with more or less developed swelling medially (34-1) and one synapomorphy: yellow, regular, relatively large patches on hemelytron present (45-1).

**Node 19**. *C. sulinus + C. simplex + C. fulvus + C. simplexoides*. This lineage is monophyletic with 70% SRS and is defined by one homoplasious character: portion between apex of clypeus and anterior margin of eye shorter than eye width in lateral view (10-1).

**Node 20**. *C. simplex + C. fulvus + C. simplexoides*. This clade is phylogenetically defined by two synapomorphies: pronotum less than two times longer than wide (28-1) and endosoma thin (50-1). This node is recovered as monophyletic by a high support value (85% SRS).

**Node 21**. *C. fulvus + C. simplexoides.* This clade is supported with 66% SRS value and one synapomorphy: basal sclerite present (53-1).

**Node 22**. *C. pilosus* clade. This assemblage is monophyletic with 59% SRS and is defined by one synapomorphy: lateral carina of pronotum well visible in dorsal view (24-1), and two homoplasious characters: punctation on pronotum moderately developed (30-1) and lateral sclerite present (52-1).

**Node 23**. This node is supported by one homoplasious feature: pale annulation on antennomere II apically present (15-1). The support values are insignificant.

**Node 24**. *C. bimaculatus* + *C. pilosus* + *C. costaricaensis + C. plectipennis* + *C. carvalhoi* + *C. cucculatus.* This clade is supported by one homoplasious character: apical sclerite present (54-0). The support values are insignificant.

**Node 25**. *C. pilosus* + *C. costaricaensis + C. plectipennis* + *C. carvalhoi* + *C. cucculatus*. This lineage is supported by one synapomorphy: apical sclerite broadened (55-1). The support values are insignificant.

**Node 26**. The monophyly of the clade composed of *C. costaricaensis + C. plectipennis* + *C. carvalhoi* + *C. cucculatus* is supported by one homoplasious character: punctation on pronotum present, moderately developed (30-1). The support values are insignificant.

**Node 27**. *C. plectipennis* + *C. carvalhoi* + *C. cucculatus*. This clade is supported by one synapomorphy: antennomere II with broad annulation apically, occupying one-quarter of antennomere II (15-1).

**Node 28**. *C. carvalhoi* + *C. cucculatus* this group is supported by one homoplasious character: lateral sclerite (LS) about twice as large as mesal sclerite (MS) (49-2).

### 3.2. Taxonomy

Subfamily: Cylapinae Kirkaldy, 1903 [1]

Tribe: Fulviini Uhler, 1886 [53]

#### 3.2.1. Genus: *Cylapocoris* Carvalho, 1954 [20] (Table 1)

*Cylapocoris* [20]: 507 (gen. nov.); [54]: 22 (key to genera), [55]: 28; [21]: 485 (diagnosis, key to species); [56]: 128 (list); [57]: 22, [58] (online catalog); [17]: 49 (list), [2]: 28 (catalog); [25]: 502 (revision, redescription, key to species). Type species: *Cylapocoris tiquinensis* Carvalho, 1954 (original designation).

**Table 1:** Checklist of the species of *Cylapocoris*.

Species
*Cylapocoris barensis* Carvalho, 1982
*Cylapocoris bimaculatus* n. sp.
*Cylapocoris brailovskyi* Wolski, 2017
*Cylapocoris brooksi* n. sp.
*Cylapocoris carvalhoi* n. sp.
*Cylapocoris castaneus* (Carvalho, 1989)
*Cylapocoris costaricaensis* Wolski, 2013
*Cylapocoris cucullatus* Wolski, 2013
*Cylapocoris fulvus* Wolski, 2013
*Cylapocoris funebris* (Distant, 1883)
*Cylapocoris laevigatus* Wolski, 2013
*Cylapocoris marmoreus* Wolski, 2013
*Cylapocoris pilosus* Carvalho, 1954
*Cylapocoris plectipennis* Wolski, 2013
*Cylapocoris salvadorensis* Carvalho, 1989
*Cylapocoris scutellatus* n. sp.
*Cylapocoris simplex* Wolski, 2013
*Cylapocoris simplexoides* n. sp.
*Cylapocoris sulinus* Carvalho and Gomes, 1971
*Cylapocoris tiquiensis* Carvalho, 1954
*Cylapocoris vittatus* Wolski, 2017

*Adcylapocoris* Carvalho 1989 [24]: 80 (gen. nov.) (syn. by Wolski [25]: [59]: 482 (list); [57]: 19, [58] (online catalog); [17]: 49 (list); [2]: 25 (catalog). Type species: *Adcylapocoris castaneus,* Carvalho 1989 (original designation).

**Diagnosis**. Recognized by the following set of features: frons weakly sloping forward (e.g., Figure 3A and Figure 6C); vertex weakly carinate (e.g., Figure 3G and Figure 6G,H); scutellum with lateral margins with single row of punctures (Figure 39 in [25]); proepimeron with distinct, deep pit near ventral margin (e.g., Figure 3C and Figure 6C); scent gland evaporative area relatively broad, triangular, portion of metepisternum bordering anterodorsal portion of evaporative area with relatively broad cavity (Figure 3C and Figure 6C); corium and clavus with a row of punctures along R + M and anal veins and medial fracture (e.g., Figure 3D and Figure 6D); tarsus with two tarsomeres, tarsomere II not divided medially (Figure 13F); pretarsal claw not toothed subapically (Figure 13F); endosoma with more or less distinctly developed medial, sclerotized, sometimes serrate lobe (ML) (e.g., Figure 10C and Figure 16C,D); distal, sclerotized part of ductus seminis inside endosoma (DSS) short, weakly longer than wide (e.g., Figure 7C,D and Figure 10C,D) sometimes distinctly abbreviated (e.g., Figure 16D and Figure 18D); endosoma either without any sclerite (e.g., Figures 23, 51, 56, 80 and 85 in [25]) or endosoma with two, more or less distinctly developed sclerites in the middle (LS and MS) and/or with single sclerite apically (AP) (e.g., Figure 7C,D and Figure 10C,D); right paramere with apical process, in dorsal view, characteristically enlarged medially, narrowed toward apex, with distinct subapical process on right hand side (e.g., Figure 4J and Figure 7H); left paramere strongly rounded, with paramere body broadened (e.g., Figure 4F,H and Figure 10F,H) and apical process, in dorsal view, widened, curved, terminated with rounded process and incised subapically (e.g., Figure 4G and Figure 16F).

**Figure 12:**
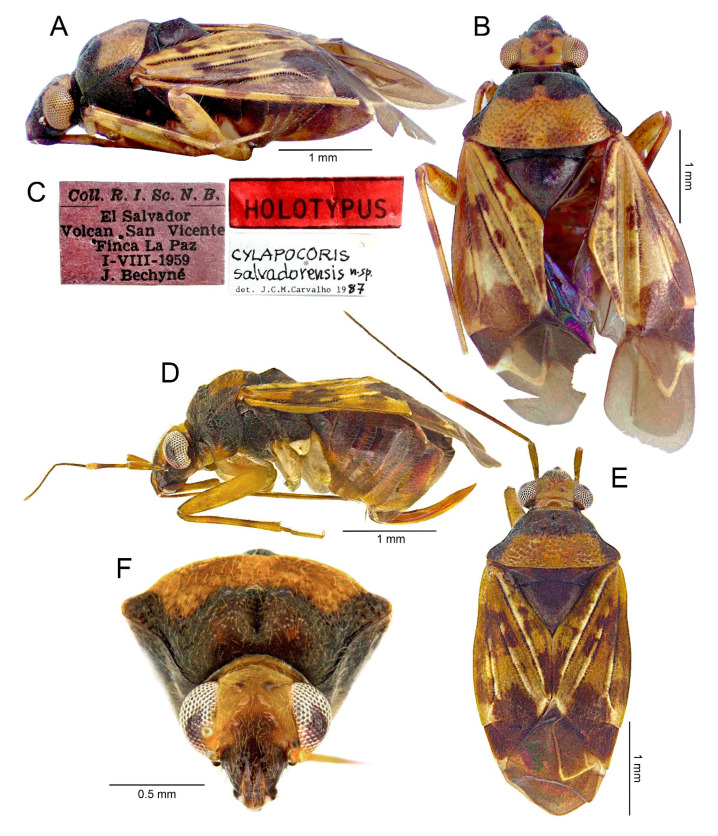
*Cylapocoris salvadorensis* Carvalho, 1989: (**A**–**C**). Holotype, (**D**–**F**). Female (Honduras): (**B**,**E**). Dorsal habitus; (**A**,**D**). Lateral view; **F**. Head and pronotum, anterior view.

**Figure 13:**
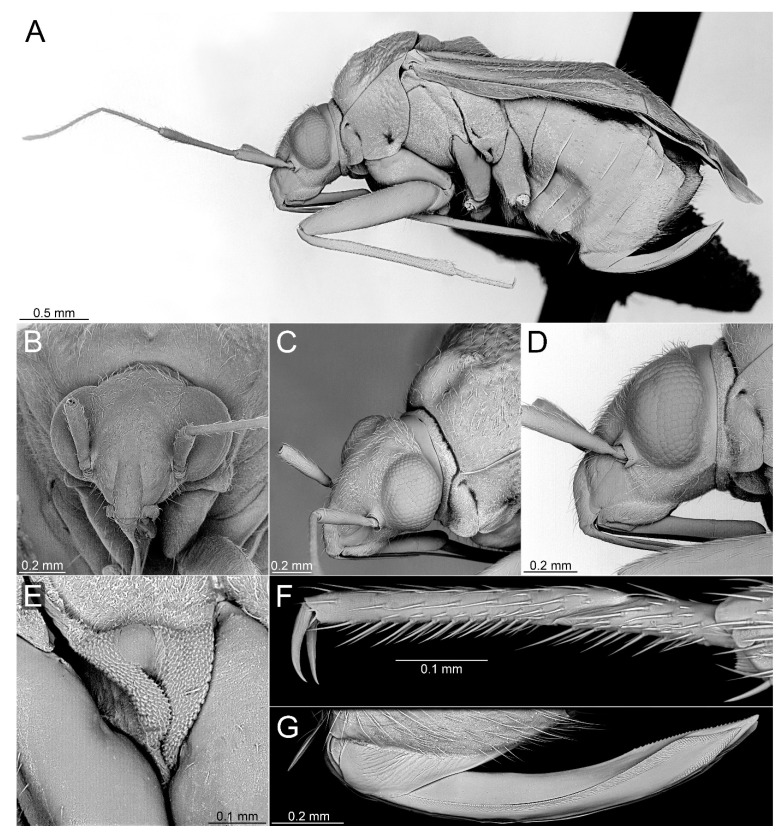
*Cylapocoris salvadorensis*, female from Honduras: (**A**). Lateral view; (**B**). Head, anterior view; (**C**). Head and pronotum, anterolateral view; (**D**). Head, lateral view; (**E**). Metathoracic scent gland efferent system; (**F**). Tarsus; (**G**). Ovipositor.

**Redescription**. *Male*. **COLORATION**. Dorsum varying from yellowish brown to dark brown, nearly black, rarely dorsum brown, mottled yellow or stramineous (e.g., Figure 3A, Figure 6A, Figure 9A and Figure 15A). *Head*. Usually dark castaneous to dark brown, rarely partially paler, yellow or ochraceous; antenna usually dirty yellowish to brown, concolorous or with more or less distinctly developed pale, whitish or yellowish annulation apically (e.g., Figure 3A,E, Figure 6A,E, Figure 9A, Figure 15A and Figure 17D). ***Thorax***. *Thoracic pleura*. Varying from yellowish brown to dark brown (e.g., Figure 3B, Figure 6B, Figure 9B, Figure 15B and Figure 17B). **TEXTURE AND VESTITURE**. Dorsal vestiture from sparse and short (e.g., Figure 3C,D) to dense and long, erect or semi–recumbent. ***Head***. Varying from rugose to moderately shining, from nearly glabrous to covered with dense, long, semi–recumbent and/or erect setae, at least partially covered with dense microsetae; antennomere I nearly glabrous basally only with several setae, remainder of antennomere covered with relatively long, reclining setae (e.g., Figure 3E–H, Figure 6E–H, Figure 9E–G and Figure 13B–D); antennomere II covered with semi–recumbent setae, rarely mixed with semi–recumbent and erect setae, vestiture usually fine, rarely thick, sometimes sparser on basal half; antennomeres III and IV thin, covered with moderately dense, semi–recumbent long setae. ***Thorax***. *Pronotum*. Ranging from matte to shining, usually more shiny than hemelytron, vestiture ranging from sparse to dense, sometimes more irregularly distributed than on pronotum, from impunctate or with shallow, sparse punctures to deeply and densely punctuate (e.g., Figure 3A–D, Figure 6A–D, Figure 9A–D and Figure 15A–C); collar densely covered with microtrichia, collar margin bordering pronotum dorsally and laterally scalloped (e.g., Figure 3C,D, Figure 6C,D, Figure 9C,D, Figure 13C and Figure 15C); calli usually punctate as remainder of pronotum, rarely impunctate or with punctures sparser than on remainder of pronotum (e.g., Figure 3A–D, Figure 6A–D, Figure 9A–D and Figure 15A–C). *Mesoscutum and scutellum*. From matte to shining (e.g., Figure 3A,B, Figure 6A,B, Figure 9A,B and Figure 12A,B) with single row of punctures on lateral margin (Figure 39 in [25]). *Thoracic pleura*. Covered with microsetae longer than on head and with sparse to dense setae (e.g., Figure 3C, Figure 6C and Figure 9C); proepimeron more or less punctate, rarely rugose (e.g., Figure 3C, Figure 6C and Figure 15C); remaining pleura usually moderately rugose, rarely somewhat shiny (Figure 3C, Figure 6C and Figure 9C). *Hemelytron*. Covered with setae ranging from sparse and short (e.g., Figure 3C,D) to dense and long (e.g., Figure 6C,D); corium and clavus with three rows of punctures, one row each along R + M and anal veins and medial fracture (e.g., Figure 3D and Figure 6D); membrane often with surface outside cells covered with minute, relatively dense setae (Figure 90 in [25]), sometimes vestiture on membrane is restricted to area situated near outer margin (Figure 91 in [25]). *Legs*. Covered with moderately long setae. ***Abdomen***. Covered with moderately dense, semi-recumbent setae. **STRUCTURE**. Macropterous. Body elongate-oval to ovoid, relatively stout (e.g., Figures 3A, 6A, 1–12 in [25]). ***Head***. Frons weakly sloping forward, longer than wide in anterior view, about as high as long in lateral view (e.g., Figure 3F–H and Figure 6F–H); vertex weakly carinate posteriorly (e.g., Figure 3G,H and Figure 6F,G); clypeus not separated from frons, clypeal base situated above ventral margin of eye (e.g., Figure 3G,H and Figure 6F,G); antennal insertion contiguous with sulcus between maxillary and mandibular plates (e.g., Figure 3G,H and Figure 6F,G); eyes contiguous with pronotal collar, eye relatively large, reniform, its ventral margin reaching gula (e.g., Figure 3G,H and Figure 6F,G); mandibular plate without sulcus posteriorly (e.g., Figure 3G,H and Figure 6F,G); antenna short, barely reaching beyond middle of body (Figure 3A and Figure 6A); antennomere I mostly cylindrical, narrowed at basal quarter (e.g., Figure 6H and Figure 9F); antennomere II usually slender, almost cylindrical, slightly incrassate (e.g., Figure 3A and Figure 6A), rarely stout or distinctly thickened at apical half (Figure 12 in [25]) or thick along entire length (Figure 2 in [25]); antennomeres III and IV thinner than antennomere II (e.g., Figure 3A, Figure 6A and Figure 9A); labium thin and long, reaching beyond metacoxae (Figure 3C and Figure 9C); segment I reaching xyphus, subdivided near medial part (Figure 3C and Figure 9C); segment II subdivided subapically (Figure 3C and Figure 9C). ***Thorax***. *Pronotum*. Collar distinct, thin, depressed, separated from remainder of pronotum by deep suture (e.g., Figure 3G and Figure 6G); calli narrow and short, from flattened to relatively convex, usually without any longitudinal sulcus between them, rarely separated by shallow sulcus, portion between collar and anterior margin of calli with more or less developed pit (e.g., Figure 3D and Figure 6D); lateral margins strongly carinate, more or less sinuate or straight; posterior margin more or less convex, sometimes nearly straight, never concave medially (e.g., Figure 3D and Figure 6D). *Mesoscutum and scutellum*. Mesoscutum well exposed; scutellum from almost flattened to distinctly convex (e.g., Figure 3D and Figure 6D). *Thoracic pleura*. Proepimeron with distinct, deep pit near ventral margin, propleural suture inclined anteriorly; mesepimeral apodeme small, oval; metathoracic spiracle slit-like without microsculpture; scent gland evaporative area relatively broad, triangular, portion of metepisternum bordering anterodorsal portion of evaporative area with relatively broad cavity; ostiolar peritreme weakly upraised, round (e.g., Figure 3C and Figure 6C). *Hemelytron*. Claval commissure about as long as scutellum; costal fracture present; cuneus well developed as wide as long; membrane with two cells, major cell large, nearly rectangular (Figure 3A and Figure 6A). *Legs*. Thin, relatively short, tarsus with two tarsomeres; tarsomere II not divided medially; pretarsal claw not toothed subapically. ***Abdomen***. *Genitalia*. Pygophore. Dorsal wall as long as ventral wall, aperture situated posteriorly (e.g., Figure 4A,E, Figure 7A,E and Figure 10A,E). Aedeagus. Theca mostly membranous, partly sclerotized apically (e.g., Figure 4B and Figure 7B); ductus seminis thin and long (e.g., Figure 4B–D and Figure 7B–D); sclerotized portion of ductus seminis (DSS) short, as long as wide (e.g., Figure 16D and Figure 18D) or longer than wide (e.g., Figure 4C and Figure 7C); endosoma often with sclerotized serrate lobe (mesial lobe—ML), either small (e.g., Figure 4C,D and Figure 10C,D) or strongly enlarged, occupying most of endosoma (e.g., Figure 16D and Figure 18D); endosoma either without sclerite (e.g., Figures 23, 56 and 80 in [25]) or with 1–3 more or less developed sclerites in middle (lateral sclerite—LS and medial sclerite—MS) and/or with single sclerite apically (apical sclerite—AP) (e.g., Figure 4C, Figure 7C,D and Figure 10C,D) sometimes endosoma also furnished with sclerite situated close to DSS (basal sclerite—BS) (Figure 18D). Left paramere. Distinctly rounded (e.g., Figure 4F,H, Figure 7F and Figure 10F,H); paramere body: broadened, with relatively dense setae, long, protruding and semi-recumbent setae situated dorsally (e.g., Figure 4F,H, Figure 7F and Figure 10F,H); apical process: broadened and curved medially, with distinct incision subapically, extreme apex rounded and narrowed (e.g., Figure 4G and Figure 10G). Right paramere. Sickle-shaped; paramere body: weakly broadened, with bundle of relatively dense, long, protruding and semi-recumbent setae situated dorsally (e.g., Figure 4I–K and Figure 7G–I); apical process: broadened in dorsal view, with distinct, subapical process situated dextrolaterally (e.g., Figure 4J, Figure 7H and Figure 10J).

**Figure 14:**
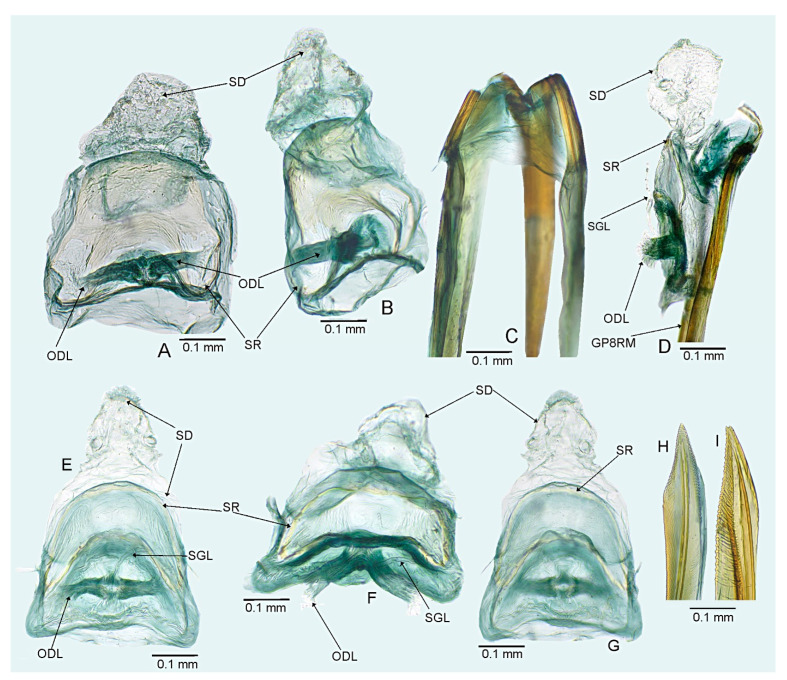
*Cylapocoris salvadorensis* Carvalho, 1989, female from Honduras (**A**–**D**). and *C. scutellatus* sp. nov. (paratype). (**E**–**I**): (**A**,**B**,**E**–**G**). Bursa copulatrix: (**A**,**E**). Dorsal view, (**B**). Dorsolateral view, (**F**). Anterodorsal view, (**G**). Ventral view; **C**. Bursa copulatrix, posterior wall; (**H**,**I**). Ovipositor: (**H**). Gonapophysis 8, (**I**). Gonapophysis 9. GP8RM = ramus of gonapophysis 8; ODL = lateral oviducts; SD = seminal depository; SGL = spermathecal gland; SR = sclerotized rings.

*Female*. Similar to male in coloration, texture, vestiture, and structure. ***Abdomen***. *Female genitalia*. Bursa copulatrix mostly membranous, thin, not extending laterally from gonapophyses 8, subtrapezoidal or semiovoid (e.g., Figure 8C, Figure 11A,B and Figure 19A), with single or paired sclerotized rings, relatively large, thick-rimmed, localized medially or laterally (e.g., Figure 5A, Figure 8D and Figure 11B,H); dorsal wall of bursa copulatrix often with large, plate-like sclerotization connected with sclerotized rings (e.g., Figure 5A, Figure 11B,H and Figure 14A); lateral oviducts contiguous, rather thin, situated posteriorly on bursa copulatrix (e.g., Figure 5A, Figure 11B,H and Figure 14A,E); spermathecal gland opens between lateral oviducts (e.g., Figure 5A and Figure 11B); vestibulum membranous, without any sclerite (e.g., Figure 8F,G, Figure 11D, Figure 14B,F,G and Figure 20B); seminal depository with ring-like structures (e.g., Figure 5A and Figure 8D); posterior wall membranous with thin sclerotization laterally (e.g., Figure 5C, Figure 8I and Figure 11C,G); apex of gonapophyses 8 and 9 sword-like (e.g., Figure 5D,E, Figure 8J,K and Figure 11E,F); apex of gonapophysis 9 dorsal margin with numerous small denticles (e.g., Figure 5E, Figure 8K and Figure 11F).

#### 3.2.2. *Cylapocoris bimaculatus* New Species (Figure 3, Figure 4 and Figure 5, Table 1)

**Diagnosis**. Recognized by the dorsum dark brown with yellow areas; antennomere II with whitish annulation apically (Figure 3A); pronotum dark with two, relatively large patches each localized sublaterally, bordering humeral angle (Figure 3A); pronotal calli moderately upraised, pit between calli deep and broad (Figure 3D,E); scutellum convex (Figure 3B,C); hemelytron dark brown with irregular yellow pattern (Figure 3A); endosoma with DSS longer than wide, incrassate (Figure 4C,D); LS broad and long, sinuate, broadened at basal half, tapering toward apex, obtuse at extreme apex (Figure 4D); MS irregularly shaped, subtriangular (Figure 4C); AP irregularly shaped, relatively broad (Figure 4D); mesal lobe (MS) present, small (Figure 4B–D); bursa copulatrix subtrapezoidal (Figure 5A); dorsal wall of bursa copulatrix with broad semiovoid sclerotization occupying anterior half; sclerotized rings situated posterolaterally, thick-rimmed, inner portion thicker, fused with anterior sclerotization (Figure 5A,B).

**Description**. **COLORATION**. Dorsal dark brown with large yellow areas (Figure 3A). ***Head*.** Mostly dark brown with two irregular yellow stripes bordering each eye, originating from posterior part of vertex and terminating near anterior insertions, clypeus weakly tinged with yellow (Figure 3A,B,E); antennomere I yellow on basal half with dark brown patch on inner surface, apical half dark brown; antennomere II mostly blackish, weakly tinged with yellow basally and with relatively broad whitish annulation apically; antennomeres III and IV blackish (Figure 3A,B,E); labial segment I dark brown yellow; segments II and III yellow; segment IV dark brown (Figure 3B). ***Thorax***. *Pronotum*. Blackish, except for two, relatively large patches, each localized sublaterally, bordering humeral angle (Figure 3A). *Mesoscutum and scutellum*. Black (Figure 3A). *Thoracic pleura*. Blackish; metepisternal scent gland evaporative area and peritreme contrastingly yellow (Figure 3B). *Hemelytron*. Yellow dark brown, rows of punctures along veins black (Figure 3A); clavus yellow brown, claval vein pale yellow along entire length, surface between row of punctures on clavus and claval suture dark brown; endocorium dark brown, broadly tinged with yellow; exocorium (embolium) mostly dark brown, with small yellow tinge anteriorly; clavus dark brown, with small dirty yellow spot on apex; membrane fuscous, venation dirty yellow (Figure 3A). *Legs*. Coxae pale yellow; femora yellow, with three brown annulations: one, broadest situated near base, one localized subapically and one apically; tibiae yellow, brown basally and with two broad brown annulations: one near base other subapically; tarsus dark yellow brown. **TEXTURE AND VESTITURE**. Dorsum moderately shining, regularly covered with relatively long and dense, semi-recumbent setae (Figure 3A–E). ***Head***. Somewhat rugose, covered with irregular, relatively long and dense, semi-recumbent and nearly erect setae (Figure 3A–E); antennomere I with a few relatively long, erect bristles and with relatively long, adpressed setae on apical two thirds, basal one third glabrous (Figure 3F–H); antennomere II covered with relatively long, semi-recumbent setae, sparse on basal half, moderately dense on apical half (Figure 3A); antennomeres III and IV mixed with long erect and shorter semi-recumbent setae (Figure 3A–C). ***Thorax***. *Pronotum*. Punctures present, rather sparse and shallow (Figure 3A–D). *Thoracic pleura*. Covered with sparse, long, semi-recumbent setae (Figure 3C). ***Abdomen***. Covered with moderately dense, long, reclining setae (Figure 3A). **STRUCTURE**. ***Head***. Antennomere II weakly incrassate (Figure 3A). ***Thorax***. Pronotal calli moderately upraised, pit between calli deep and broad (Figure 3A–D). *Scutellum*. Moderately convex (Figure 3A–D) ***Abdomen***. *Genitalia*. Aedeagus. Endosoma with DSS longer than wide, incrassate; LS broad and long, sinuate, broadened at basal half, tapering toward apex, obtuse at extreme apex; MS irregularly shaped, subtriangular; AP irregularly shaped, relatively broad; mesal lobe (MS) present, small (Figure 4B–D). Left paramere. Paramere body: strongly broadened; apical process: lateral view: weakly curved; dorsal view: subapical incision distinct (Figure 4F–H). Right paramere. Paramere body: inner margin weakly sinuate in lateral view; apical process: lateral view: straight with obtuse extreme apex; dorsal view: moderately rounded at extreme apex, medial process (MP) large, rounded, left lateral margin distinctly curved (Figure 4I–K).

*Female*. Similar to male in coloration, texture, vestiture, and structure. *Genitalia*. Bursa copulatrix subtrapezoidal (Figure 5A); dorsal wall of bursa copulatrix with broad semiovoid sclerotization occupying anterior half; sclerotized rings situated posterolaterally, thick-rimmed, inner portion thicker, fused with anterior sclerotization (Figure 5A,B).

**Measurements**. (♀/♂, *: holotype measurements): *Body*. Length: 4.1–4.8/3.7*, width 2.2–1.7/1.6*. *Head*. Length: 0.64–0.80/0.64*, width: 0.80–0.90/0.82*, interocular distance 0.40–0.42/0.40*. *Antenna*. Length of antennomere I: 0.38–0.42/0.43*, II: 1.02–1.18/1.25*, III: 0.53–0.58/0.66*, IV: 0.80–0.88/missing in holotype. *Labium*. Length of segment I: 0.62–0.75/0.63*, II: 0.62–0.75/0.66*, III: 0.68–0.7/0.75*, IV: 0.50–0.65/0.55*. *Pronotum*. Length: 0.75–1.0/0.79*, width of anterior margin: 0.7–0.85/0.61*, length of lateral margin: 0.75–0.95/0.79*, width of posterior margin: 1.4–1.8/1.45*.

**Etymology**. The specific name is derived from the Latin “bi”, meaning two, and “macula”, meaning spot, and is used to denote the presence of the two yellow patches on posterior lobe of pronotum.

**Biology**. Unknown.

**Distribution**. Costa Rica (Guanacaste, Puntarenas), Panama (Chriqui) (“2” in Figure 22).

**Remarks**. This species is most similar to *C. salvadorensis* in sharing the antennomere II with whitish annulation apically (Figure 3A and Figure 12E), the convex scutellum (Figure 3B,C and Figure 12A,D), the hemelytron dark brown with large irregular yellow pattern including yellow line along claval vein (Figure 3A, Figure 12B), the moderately upraised pronotal calli (Figure 3D, E and Figure 13C), and the presence of anterior sclerotization on bursa copulatrix fused with sclerotized rings (Figure 5A,B and Figure 14A). It is clearly distinguished by the lack of large yellow patch on the posterior lobe of pronotum (only two sublateral yellow patches, each bordering humeral angles, are present) (Figure 3A) and the shorter anterior sclerotization on anterior part of bursa copulatrix dorsal wall (Figure 5A).

**Type material**. Holotype ♂: ‘Costa Rica: Puntarenas Province, Las Alturas Biological Station, 1660 m, 08°56′17″ N, 82°50′01″ W, 31–V–2004, J.S. Ashe, Z. Falin, I. Hinojosa. Ex: pyrethreum fogging free covered with fleshy white polypores; CR1AFH04 065′ (**KUNHM**). Paratypes: 1♀: Panama: Chiriqui 27.7 km, W Volcan Hartmann’s Finca, 1450 m 8°51′48″ N, 82°44′36″ W; 17 Jun 1996; J. Ashe, R. Brooks, PAN1AB96 168; ex: fungusy log; SM00427 749; 1♀: the same data except for: J. Ashe and R. Brooks#223, ex: fogging fungusy log; KU Loan 2017; 1♀: Costa Rica: Guanacanaste Palo Verde Biological Station Sendero Natural La Venada, 10 m 10°20′56″ N, 85°19′53″ W 16 July 2000; J. Ashe, R. Brooks, Z. Falin, CR1ABF00 148, ex: fogging fungus-covered log (**KUNHM**).

#### 3.2.3. *Cylapocoris brooksi* New Species (Figure 6, Figure 7 and Figure 8, Table 1)

**Diagnosis**. Recognized by the dorsum covered with dense, long semi-recumbent and erect setae (Figure 6A–E); antennomere II entirely yellow to dark yellow, sometimes tinged with brown (Figure 6A,B); posterior lobe of pronotum almost entirely ochraceous, except blackish lateral margin (Figure 6A); endosoma with distal sclerotized portion of ductus seminis caliciform, longer than wide; lateral sclerite (LS) small, situated subapically on endosoma, arcuate, weakly tapering; medial sclerite (MS) small, about three times smaller than MS, irregularly shaped (Figure 7B–D); dorsal wall of bursa copulatrix with single large, thick-rimmed, semi-ellipsoid sclerotized ring occupying most of bursa copulatrix, its anterior margin strongly convex, posterior margin strongly concave (Figure 8C,D,F,G).

**Description**. **COLORATION**. Ochraceous with dark ochraceous areas (Figure 6A). ***Head***. Ochraceous with small dark brown areas (Figure 6A,B,E); antenna ochraceous dark brown; antennomeres I and II ochraceous, antennomere I with dark brown annulation near base and small dark brown tinge apically; antennomere II with narrow dark brown tinge basally and apically; antennomeres III and IV dark brown except brownish basal portion of antennomere III (Figure 6A,B); labium ochraceous, sometimes tinged with dark brown. ***Thorax***. *Pronotum*. Mostly ochraceous, calli area dark ochraceous brown to dark brown, lateral margin blackish (Figure 6A,B). *Mesoscutum and scutellum*. Dark ochraceous (Figure 6A,B); scutellum sometimes with ochraceous patch apical part. *Thoracic pleura*. Propleuron dark brown; remaining pleura blackish; metepisternal scent gland evaporative area partly dirty yellow anteriorly, dark brown posteriorly, peritreme dark ochraceous (Figure 6A). *Hemelytron*. Dark ochraceous to dark brown; membrane sandy to dark brown, venation mostly dirty yellow (Figure 6A). *Legs*. Mostly ochraceous; base of coxae often fuscous; fore coxa sometimes entirely fuscous; tibiae and femora sometimes weakly tinged with dark brown. ***Abdomen***. Mostly dark brown with irregular, dark reddish and dark ochraceous areas (Figure 6B). **TEXTURE AND VESTITURE**. Dorsum mixed with long, dense, golden, semi-recumbent and erect setae (Figure 6A–E). ***Head***. Weakly rugose, covered with long semi-recumbent and erect setae, vestiture on frons sparser than on remainder of head; (Figure 6F–H); antennomere I with a few relatively long, erect bristles and with relatively long, adpressed setae on apical two thirds, basal one third glabrous (Figure 6G,H); antennomere II covered with relatively long, semi-recumbent setae, sparse on basal half, moderately dense on apical half (Figure 6A). ***Thorax***. *Pronotum*. Moderately shining; punctation dense and shallow (Figure 6A,D,E). *Mesoscutum and scutellum*. Weakly rugose (Figure 6A,D). *Thoracic pleura*. Covered with sparse, erect, relatively long setae; propleuron shallowly punctate (Figure 6B,C). ***Abdomen***. Covered with long, reclining setae (Figure 6B). **STRUCTURE**. ***Thorax***. *Pronotum*. Calli weakly upraised, pit between calli shallow and narrow (Figure 6D, E). *Scutellum*. Weakly convex (Figure 6A–C). ***Abdomen***. *Genitalia*. Aedeagus. Endosoma relatively narrow (Figure 7B–D); theca moderately sclerotized (Figure 7B); distal sclerotized portion of ductus seminis inside endosoma caliciform, longer than wide (Figure 7C,D); lateral sclerite (LS) small, situated subapically on endosoma, arcuate, weakly tapering (Figure 7C,D); medial sclerite (MS) small, about three times smaller than MS, irregularly shaped (Figure 7C,D). Left paramere. Paramere body: strongly broadened (Figure 7F); apical process: lateral view: weakly curved; subapical incision distinct. Right paramere. Paramere body: inner margin weakly sinuate in lateral view (Figure 4G,I); apical process: lateral view: weakly curved with acute extreme apex; dorsal view: acute at extreme apex, medial process (MP) large, rounded (Figure 4H).

*Female*. Similar to male in color, texture, vestiture, and structure. ***Abdomen***. *Genitalia*. Bursa copulatrix semiovoid (Figure 8C,D); dorsal wall of bursa copulatrix with single large, thick-rimmed, semi-ellipsoid sclerotized ring occupying most of bursa copulatrix, its anterior margin strongly convex, posterior margin strongly concave (Figure 8C,D,F,G).

**Measurements**. (♀/♂, *: holotype measurements): *Body*. Length: 3.9–4.3/3.35–3.5 (3.4*), width 1.65–1.95/1.5–1.7 (1.6*). *Head*. Length: 0.68–0.73/0.6*–0.65, width: 0.8–0.85/0.75–0.85 (0.8*), interocular distance 0.4–0.42/0.30*–0.35. *Antenna*. Length of antennomere I: 0.3–0.4/0.33–0.4 (0.3*), II: 0.95–0.98/0.9*–0.95, III: 0.8/0.68–0.73 (0.6*), IV: –/0.97–1.0. *Labium*. Length of segment I: 0.62–0.63/053*, II: 0.58–0.63/–, III: –/0.58, IV: 0.5–0.58/–. *Pronotum*. Length: 0.65–0.75/0.6–0.70*., width of anterior margin: 0.6–0.7/0.6*–0.65, length of lateral margin: 0.7–0.8/0.6–0.7*, width of posterior margin: 1.4–1.6/1.3–1.4*.

**Etymology**. The species is named in honour of its collector, R. Brooks.

**Biology**. Unknown.

**Distribution**. Peru (Madre de Dios) (“3” in Figure 22).

**Remarks**. *C. brooksi* is most similar to *C. brailovskyi* in sharing the shallow punctation of pronotum. With *C. brailovskyi*, *C. costaricaensis*, *C. bimaculatus*, *C. pilosus*, *C. plectipennis*, *C. carvalhoi*, and *C. cucculatus* it shares the presence of lateral sclerite of endosoma (LS). It can, however, be distinguished by the ochraceous dorsum, the antennomere II without pale annulation apically, and the shape of the female genitalia.

**Type material**. Holotype ♂: ‘Peru: Madre de Dios, Cocha Cashu Biological Station, Manu National Park, 350 m, 11°53′45″ S, 71°24′24″ W, 18 October 2000; R. Brooks, Peru 1B 00 024, ex: fungus-covered log’ (**KUNMHM**). Paratypes: 2♀♀ and 3♂♂: the same data as for holotype except for 1♀: ‘SM0271 273 KUNHM-ENT’; 1♂: ‘SM0267 909 KUNHM-ENT’; 1♂: ‘SM0267 908 KUNHM-ENT’; 1♀: ‘SM0267 963 KUNHM-ENT’; 1♂: ‘SM0267 974 KUNHM-ENT’; 1♂: Peru: ‘Madre de Dios, Pakitza Biological Station, 317 m Castanal Trail, Reserved Zone, Manu National Park, 11°56′41″ S, 71°17′0″ W, 16 October 2000, R. Brooks, Peru 1B 00 016, ex: fungus-covered log; SM0268 370 KUNHM-ENT’ (**KUNHM**).

#### 3.2.4. *Cylapocoris carvalhoi* New Species (Figure 9 and Figure 10, Table 1)

**Diagnosis**. Recognized by the antennomere II dark brown with apical one third with pale annulation (Figure 9A); pronotum with large and deep punctures (Figure 9A,D); hemelytron uniformly dark brown without any patches (Figure 9A); sclerotized portion of ductus seminis inside endosoma caliciform, longer than wide (Figure 10C,D); medial lobe small, sub-ovoid (Figure 10C,D); lateral sclerite (LS) small, weakly curved, broadened at basal half, tapering, moderately pointed; medial sclerite (MS) about as large as LS, irregularly shaped (Figure 10C,D); apical sclerite (AS) present, relatively short and narrow, beak-shaped (Figure 10C,D).

**Description**. **COLORATION**. Dorsum dark brown with black and dark yellow areas. ***Head***. Dark brown, vertex with two large dark yellow tinges bordering margin of each eye; antenna dark brown with dark yellow and orange areas; antennomere I dark brown broadly tinged with dark yellow basally; antennomere II with apical one third with orange annulation; antennomeres III and IV dark brown, except for narrow yellow annulation of antennomere IV apically; labium dark brown. ***Thorax***. *Pronotum*. Blackish; collar dark ochraceous. *Mesoscutum and scutellum*. Dark brown. *Thoracic pleura*. Blackish, metathoracic scent gland evaporative area contrastingly yellow. *Hemelytron*. Dark brown. *Legs*. Coxae dirty yellow; other segments missing in the examined specimens. ***Abdomen***. Dark brown; ventrolateral portions of segments V–VIII and entire pygophore ochraceous. **TEXTURE AND VESTITURE**. Dorsum shining, covered with moderately dense and long, semi-recumbent setae. ***Head***. Somewhat rugose, covered with rather short, moderately dense setae, sparser on frons. ***Thorax***. *Pronotum*. Punctures present, large and deep. *Thoracic pleura*. Covered with sparse, semi-recumbent setae. **STRUCTURE**. ***Head***. Antennomere II weakly incrassate. ***Thorax***. *Pronotum*. Calli flat, pit between them shallow and narrow. ***Abdomen***. Genitalia. Sclerotized portion of ductus seminis inside endosoma caliciform, longer than wide (Figure 10C,D); medial lobe small, sub-ovoid (Figure 10C,D); lateral sclerite (LS) small, weakly curved, broadened at basal half, tapering, moderately pointed; medial sclerite (MS) about as large as LS, irregularly shaped (Figure 10C,D); apical sclerite (AS) present, relatively short and narrow, beak-shaped (Figure 10C,D). Left paramere. Paramere body: strongly broadened (Figure 10F,H); apical process: lateral view: straight; subapical incision distinct (Figure 10F–H). Right paramere. Paramere body: inner margin weakly sinuate in lateral view (Figure 10I,K); apical process: lateral view: curved with obtuse extreme apex; dorsal view: moderately pointed at extreme apex, medial process (MP) weakly developed, rounded (Figure 10J).

**Measurements**. Holotype ♂: *Body*. Length: 3.37, width: 1.41. *Head*. Length: 0.32, width: 0.82, interocular distance: 0.35. *Antenna*. Length of antennomere I: 0.44, II: 1.23, III: 0.67, IV: missing. *Labium*. Length of segment I: 0.52, II: –(impossible to measure), III: –, IV: 0.52. *Pronotum*. Length: 0.72, width of anterior margin: 0.61, length of lateral margin: 0.66, width of posterior margin: 1.32.

**Etymology**. Named after the late José Cândido de Melo Carvalho for his many outstanding contributions to miridology.

**Biology**. Unknown.

**Distribution**. Costa Rica (Puntarenas) (“2” in Figure 22).

**Remarks**. *C. carvalhoi* is most similar to *C. costaricaenis*, *C. cucculatus*, *C. funebris*, and *C. plectipennis* in sharing the pronotum with deep and large punctures and hemelytron uniformly dark brown without any pale patches. It can, however, be distinguished by the shape of the endosomal sclerites and parameres.

**Type material**. Holotype ♂: ‘Costa Rica: Puntarenas Province, Golfito, 10–200 m, 28 May 1993, J.S. and A. Ashe #071, ex: mushrooms’ **(KUNHM**).

#### 3.2.5. *Cylapocoris cucculatus* Wolski, 2013 (Figure 11A–F, Table 1)

**Figure 15:**
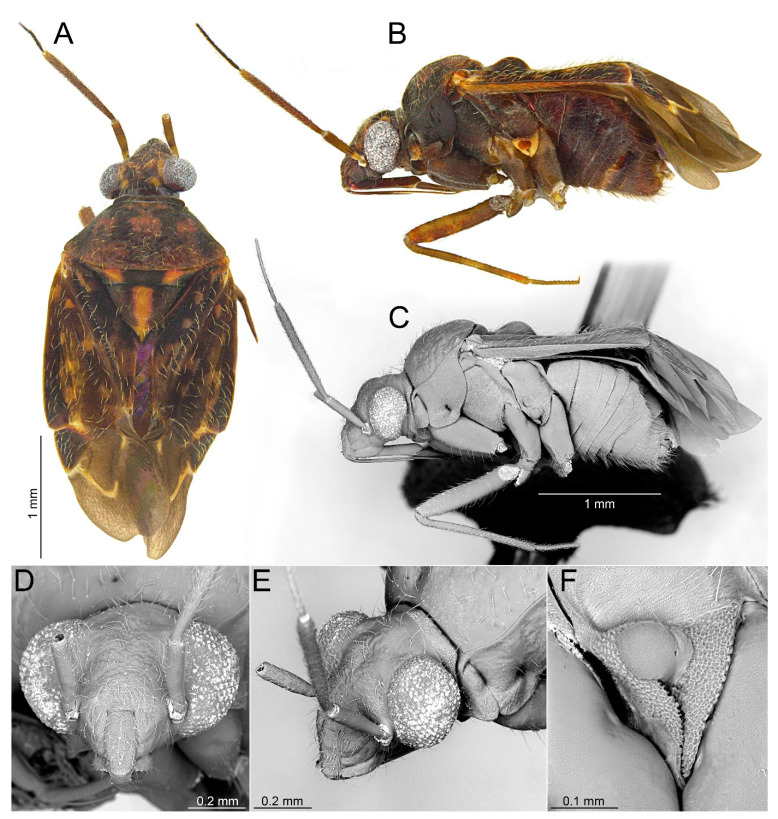
*Cylapocoris scutellatus* sp. nov., holotype: (**A**). Dorsal view; (**B**,**C**). Lateral view; (**D**). Head, anterior view; (**E**). Head, anterolateral view; (**F**). Metathoracic scent gland efferent system.

*Cylapocoris cucculatus* Wolski, 2013 [25]: 504, 510, Figures 3, 15, 40–44 (n. sp.)

**Diagnosis**. See [25].

*Female*. *Genitalia*. Bursa copulatrix semiovoid (Figure 11A,B); dorsal wall of bursa copulatrix with large, crescent-like sclerotization occupying anterior half of genital chamber; sclerotized rings paired, each situated posterolaterally, thick-rimmed, ovoid, large, anterior edge reaching middle of bursa copulatrix, fused with anterior sclerotization (Figure 11B).

**Distribution**. Costa Rica, Honduras (“5”, “6” and “7” in Figure 22).

**Type material**. Holotype ♂: COSTA RICA: Heredia: La Selva Field Sta. near Puerto Viejo, 21–28 March 1988; W. E. Steiner, J. M. Hill, J. M. Swearingen; J. M. Mitchell (**USNM**).

**Additional material examined**. 1♂: Costa Rica: Guanacaste Patilla Biological Station, 610 m, 10°59′22″ N, 85°25′33″ W (**KUNHM**); 1♀: Honduras: Ocotepeque, 24 E Ocotepeque El Güisayote, 16 VI 1994, 2170 m, 14°25′N, 89°04′W, J. Ashe, R. Brooks, #118; KU Loan 2017 (**KUNHM**).

#### 3.2.6. *Cylapocoris funebris* (Distant, 1883) (Figure 11G–I, Table 1)

*Camus funebris* Distant 1883 [60]: 288 (n. sp.)

*Carmelus* [sic] *funebris*: [61] (catalog)

*Cylapocoris funebris*: [22]: 56, Figure 5 (discussion of holotype); [62]: 797 (discussion of holotype); [57]: 22 (catalog); [2]: 28 (catalog); [25]: 504, 513, Figures 5 and 17 (diagnosis, redescription).

**Diagnosis**. See [25].

*Female*. *Genitalia*. Bursa copulatrix semicircular (Figure 11G,H); dorsal wall of bursa copulatrix with narrow, crescent-like sclerotization anteriorly (Figure 11H), with two large thick-rimmed, sub-ovoid, sclerotized rings, each situated posterolaterally, anterior edge reaching weakly beyond medial part, fused with anterior sclerotization (Figure 11H).

**Distribution**. Panama, Costa Rica (Guanacasta) (“2” and “6” in Figure 22).

**Type material**. Holotype ♀: Bugaba, Panama, Champion; Carnus funebris Dist.; type [round label]; female symbol; BMNH (E) #909457 (**BMNH**).

**Additional examined material**. 1♀: Costa Rica: Guanacasta Prov., Las Cañas, Finca La Taboga, 17–27 June 1969, Toby Schuh, Janet Crane (**AMNH**); 1♀: Costa Rica: Guanacaste Patilla Biological Station, 610 m 10°59′22″ N, 85°25′33″ W, 13 July 2000; J. Ashe, R. Brooks, Z. Falin, CR1ABF00 111, ex: fogging fungus-covered log; SM0 201 257 KUNHM-ENT (**KUNHM**).

#### 3.2.7. *Cylapocoris salvadorensis* Carvalho, 1989 (Figure 12, Figure 13 and Figure 14A–D, Table 1)

*Cylapocoris salvadorensis* Carvalho 1989 [63]: 266, Figure 8 (n. sp.); [59]: 490 (list); [57]: 22 (catalog); [2]: 28 (catalog); [25]: 505, 521 (key to species of *Cylapocoris*, discussion).

**Diagnosis**. Recognized by the posterior lobe of pronotum being yellow except for black humeral angles (Figure 12B,E); antennomere II with whitish annulation apically (Figure 12D,E); hemelytron dark brown with irregular large yellow pattern including yellow line along claval vein (Figure 12B,E); dorsal wall of bursa copulatrix with broad subtrapezoidal sclerotization anteriorly occupying most of it (Figure 14A,B), with two thick-rimmed sclerotized rings situated posterolaterally, fused with anterior sclerotization (Figure 14A,B).

**Redescription**. *Female*. **COLORATION**. Dorsum yellow with large black and dark brown areas (Figure 12B,E). ***Head***. Vertex yellow with irregular tinge medially; frons with posterior and lateral portions yellow (Figure 12B,E,F), remainder of head black (Figure 12B,E,F); clypeus with yellowish tinge basolaterally (Figure 12F); antennomeres I and II fuscous (Figure 12E); antennomere II with narrow whitish annulation apically (Figure 12E); antennomeres III and IV black (Figure 12E); labium yellowish, weakly tinged with dark brown (Figure 12D). ***Thorax***. *Pronotum*. Collar dark yellowish black; calli, lateral margin, and humeral angle black, surface between posterior portions of calli sometimes with yellow spot; posterior lobe yellow (Figure 12B,E). *Mesoscutum and scutellum*. Black (Figure 12B,E). *Thoracic pleura*. Mostly black; propleuron with yellowish tinge ventrally; evaporative areas and peritreme yellow to dark yellow (Figure 12A,D). *Hemelytron*. Clavus yellow with irregular brown pattern on basal two thirds, apical one third brown, claval vein pale yellow along entire length, row of punctures along claval vein brown, surface between row of punctures on clavus and claval suture yellow; endocorium yellow with irregular brown pattern from area near base to medial part, apex with broad, brown patch; exocorium almost entirely brown, narrowly yellow basally and apically, medial fracture pale yellow along entire length, row of punctures along medial fracture and R + M veins dark brown; cuneus dark brown except for small yellow spot apically; membrane fuscous, its minor cell with pale yellow venation, major cell venation fuscous from basal to subapical region, apical part yellow (Figure 12B,E). *Legs*. Coxae yellow, sometimes weakly tinged with brown; femora yellow, with relatively large, brown tinges; tibiae yellow, base with narrow brown patch and with two broad, brown annulations: one situated near base and other near middle; tarsus yellow (Figure 12A,D). **TEXTURE AND VESTITURE**. Dorsum covered with moderately dense, semi-recumbent setae (Figure 12A and Figure 13A). ***Head***. Smooth, covered with moderately dense, semi-recumbent setae (Figure 13B,C,D); vertex with shining narrow and short transverse area on vertex, between eyes, and two long oblique area originating from posterior part of vertex and terminating at antennal insertion, clypeus, maxillary and mandibular plates and anterior part of buccula shiny, glabrous without microtrichia and setae (Figure 13B–D); antennomere II with relatively long, semi-recumbent setae, basal half with sparse vestiture, apical half with relatively dense setae (Figure 13A). ***Thorax***. *Pronotum*. With dense and relatively shallow punctures. *Legs*. Covered with moderately dense, semi-recumbent setae. ***Abdomen***. Covered with moderately dense reclining setae. **STRUCTURE**. ***Head***. Antennomere II weakly incrassate. ***Thorax***. *Pronotum*. Calli moderately upraised, pit between them relatively broad. *Scutellum*. Convex. ***Abdomen***. *Genitalia*. Bursa copulatrix subtrapezoidal (Figure 14A); dorsal wall of bursa copulatrix with broad subtrapezoidal sclerotization anteriorly occupying most of it (Figure 14A,B), with two thick-rimmed sclerotized rings situated posterolaterally, fused with anterior sclerotization (Figure 14A,B).

*Male.* Unknown.

**Measurements**. (♀, *: holotype measurements): *Body*. Length: 3.75–4.8*, width 1.5–1.8*. *Head*. Length: 0.6*–0.68, width: 0.78–0.9*, interocular distance 0.38–0.43 (0.4*). *Antenna* (missing in holotype). Length of antennomere I: 0.42, II: 1.0–1.02, III: 0.58, IV: 4.0. *Labium*. Length of segment I: 0.62, II: 0.6, III: 0.7, IV: 0.53. *Pronotum*. Length: 0.7–0.8*, width of anterior margin: 0.7–0.75, length of lateral margin: 0.7, width of posterior margin: 1.4–1.6*.

**Distribution**. Salvador (San Vincente) [63]; Honduras (Cortés, Atlántida) (this paper) (“11” and “12” in Figure 22).

**Remarks**. *C. salvadorensis* is most similar to *C. bimaculatus* (see Remarks under *C. bimaculatus* for further details).

**Type material**. Holotype ♀: Coll. R. I. Sc. N. B., El Salvador, Volcan San Vincente, Finca La Paz, I–VIII–1959, J. Bechyné (**ISNB**).

**Additional material examined**. 7♀♀: Honduras: Atlantida Lancetilla Botanical Garden, Tela, 10 m, 22 June 1994, 15°46′ N, 87°27′ W, J. Ashe, R. Brooks # 181, ex: fogging fungusy log; KU Loan 2017; 1♀: Honduras: Santa Barbara, La Fe, Finca La Roca, 5.3 km S Peña Blanca, 14°57′ N, 88°02′ W, 740 M, 19 VI 1994, Brooks, Ashe, #160, ex: fog fungusy log (**KUNHM**).

#### 3.2.8. *Cylapocoris scutellatus* New Species (Figure 15 and Figure 16, Table 1)

**Figure 16:**
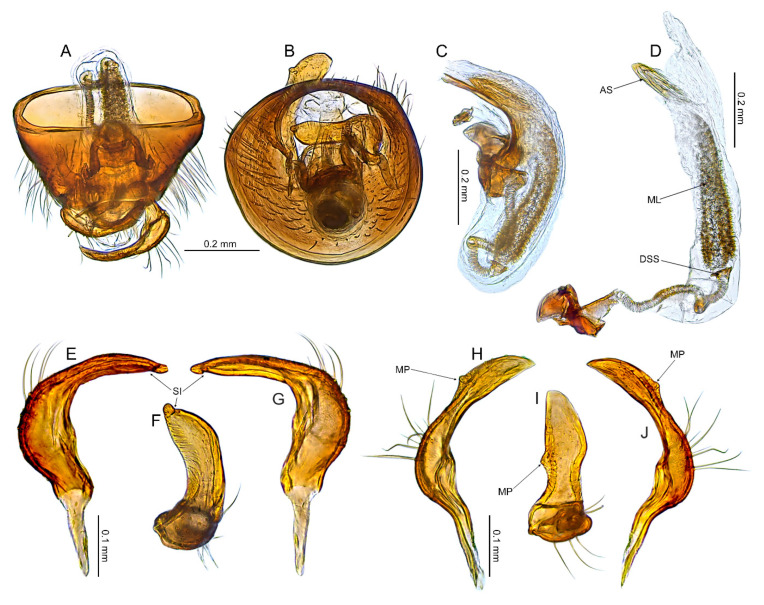
*Cylapocoris scutellatus* sp. nov., holotype: (**A**,**B**). Pygophore: (**A**). Dorsal view, (**B**). Caudal view; (**C**). Aedeagus with theca, left lateral view; (**D**). Aedeagus, theca removed, left lateral view; (**E**–**G**). Left paramere: (**E**). Right lateral view, (**F**). Apical process, dorsal view, (**G**). Left lateral view; (**H**–**J**). Right paramere: (**H**). Right lateral view, (**I**). Apical process, ventral view, (**J**). Left lateral view. AS = apical sclerite; DSS = sclerotized part of ductus seminis inside endosoma; ML = mesial lobe; MP = medial process; SI = subapical incision.

**Diagnosis**. Easily recognized by the ovoid body (Figure 15A); antennomere II entirely dark brown (Figure 15A,B); scutellum convex with broad longitudinal stramineous reddish framed stripe medially originating from base and terminating subapically (Figure 15A); hemelytron dark brown mottled with stramineous and ivory areas (Figure 15A); endosoma thin, sclerotized part of ductus seminis inside endosoma incrassate, as long as wide; medial lobe (ML) well developed, occupying most of endosoma; apical sclerite present short cylindrical obtuse (Figure 16C,D); bursa copulatrix semiovoid, dorsal wall with single sclerotized ring, thick-rimmed, semi-ellipsoid large, occupying anterior half of genital chamber, its anterior margin strongly convex, posterior margin strongly concave (Figure 14E–G).

**Description**. *Male*. **COLORATION**. Dorsum dark brown with stramineous, reddish, and dark castaneous areas (Figure 15A,B). ***Head***. Mostly dark brown; vertex and frons with regular stramineous pattern (Figure 15A,B); antennomere I dark stramineous with dark brown annulation near base (Figure 15A,B); antennomere II from dark stramineous to fuscous, sometimes with whitish annulation apically, antennomeres III and IV dark brown, almost blackish; labium castaneous (Figure 15B). ***Thorax***. *Pronotum*. Dark brown with large castaneous tinge (Figure 15A,B). *Mesoscutum and scutellum*. Dark brown; mesoscutum with two relatively large stramineous reddish framed patches, each situated sublaterally (Figure 15A); scutellum with broad longitudinal dark yellow reddish framed stripe medially originating from base and terminating subapically, apex with small triangular patch bordering longitudinal stripe (Figure 15A). *Thoracic pleura*. Dark brown; proepisternum dark yellow ventrally; metathoracic scent gland evaporative area yellow; peritreme ochraceous (Figure 15B). *Hemelytron*. Dark brown with stramineous and ivory areas; clavus dark brown with irregular ivory-colored pattern; endocorium dark brown with stramineous mottling; exocorium (embolium) dark brown with small stramineous patch subanteriorly and narrow, irregular stramineous stripe from middle of medial fracture to inner angle of cuneus; lateral margin stramineous along entire length; membrane dark grey, cell venation yellow except dark gray inner margin of both cells (Figure 15A,B). *Legs*. Coxae dark brown; fore- and meta femora dark brown with irregular stramineous tinge; fore- and meta tibia dark brown with relatively broad stramineous annulations: one situated medially and other apically; middle leg missing in the examined specimens. ***Abdomen***. Dark brown (Figure 15B). **TEXTURE AND VESTITURE**. Dorsum weakly shining, covered with irregularly distributed, relatively long, sparse, erect and semi-recumbent golden setae (Figure 15A–C). ***Head***. Head rugose, covered with irregularly distributed relatively long, moderately dense setae, sparser on vertex and frons (Figure 15D,E); antennomere I covered with sparse relatively long adpressed setae on apical half, glabrous on basal half (Figure 15C); antennomere II mixed with relatively dense, relatively long reclining and semi-recumbent setae, basal portion narrowly glabrous (Figure 15C). ***Thorax***. *Pronotum*. Setae shorter and more irregularly distributed than on hemelytron (Figure 15A,C); punctures present, small, shallow and densely distributed (Figure 15A,C). *Mesoscutum and scutellum*. Vestiture sparser than on pronotum (Figure 15A). *Thoracic pleura*. Covered with relatively long, sparse, semi-recumbent setae; proepimeron nearly glabrous, setae present only on dorsal and ventral narrow portions (Figure 15B,C). **STRUCTURE**. Body ovoid (Figure 15A). ***Head***. Antennomere II cylindrical (Figure 15A). ***Thorax***. *Pronotum*. Calli flat, indistinct (Figure 15A). *Scutellum*. Convex (Figure 15A–C). ***Abdomen***. *Genitalia*. Aedeagus. Endosoma thin, sclerotized part of ductus seminis inside endosoma incrassate, as long as wide; medial lobe (ML) well developed, occupying most of endosoma; apical sclerite present short cylindrical obtuse (Figure 16C,D). Left paramere. Paramere body: strongly broadened (Figure 16E–G); apical process: lateral view: straight; subapical incision shallow (Figure 16F). Right paramere. Paramere body: inner margin weakly sinuate in lateral view (Figure 16H,J); apical process: lateral view: semi-ellipsoid; dorsal view: broad, obtuse, medial process (MP) well developed, rounded (Figure 16I).

*Female*. Similar to male in coloration, texture, vestiture, and structure. ***Abdomen***. *Genitalia*. Bursa copulatrix semiovoid, dorsal wall with single sclerotized ring, thick-rimmed, semi-ellipsoid, large, occupying anterior half of genital chamber, its anterior margin strongly convex, posterior margin strongly concave (Figure 14E–G).

**Measurements**. (♀/♂, *: holotype measurements): *Body*. Length: 3.9/3.15–3.4*, width 1.7/1.4,5*. *Head*. Length: 0.75/0.65*, width: 0.85/0.80*–0.82, interocular distance 0.45/0.35–0.4*. *Antenna*. Length of antennomere I: 0.4/0.38–0.4*, II: 1.12/1.0*, III: 0.55/0.5*, IV: missing in the female specimen/0.78–1.1. *Labium*. Length of segment I: 0.7/0.62–0.63, II: 0.5/0.78–0.8, III: immeasurable in the female specimen/0.5, IV (immeasurable in the examined specimens). *Pronotum*. Length: 0.85/0.7*–0.85, width of anterior margin: 0.7/0.7*, length of lateral margin: 0.75/0.6–0.75, width of posterior margin: 1.45/1.2–1.35 (1.3*).

**Etymology**. The specific epithet “scutellatus” is used to denote the dark scutellum with broad longitudinal dark yellow reddish framed stripe medially.

**Biology**. Unknown.

**Distribution**. Ecuador (Pichincha) (“13” in Figure 22).

**Remarks**. *C. scutellatus* is most similar to *C. marmoreus* in the small ovoid body, the convex scutellum, the hemelytron with mottled coloration, and by the medial lobe occupying most endosoma. It can, however, be distinguished by the coloration of scutellum and the shape of the male genitalia.

**Type material**. Holotype ♂: Ecuador: Pichincha, Pedro Vicente Maldonado, 3.5 km N, 530 m, 0°6′44″ N, 79°3′21″ W, 29 March 1999, R. Brook, ECU1B99 066, ex: fungus, mushroom, polypore, white (**KUNHM**). Paratypes: 2♂♂, 1♀: the same data as for the holotype except for: ♂: SM0 156 182 KUNHM-ENT, ♂: SM0 156 185 KUNHM-ENT, ♀: SM0 156 201 KUNHM-ENT (**KUNHM**).

#### 3.2.9. *Cylapocoris simplex* Wolski, 2013 (Figure 19A–E, Table 1)

*Cylapocoris simplex* Wolski, 2013 [25]: 505, 521, Figures 10, 21, 72–84 (n. sp.)

**Diagnosis**. See [25].

*Female*. *Genitalia*. Bursa copulatrix semiovoid, membranous without any sclerotizations or sclerotized rings (Figure 19A,B); posterior wall of bursa copulatrix with interramal sclerite broad, subtriangular (Figure 19C).

**Type material**. Holotype ♂: Ecuador, Orellana Prov. [labeled Napo in error], Res. Ethnica Waorani, 1 km. S, Onkone Gare Camp, Trans. Ent., 29 June 1994, 220 m, 00°39′10″ S, 76 °26′00″ W, T.L. Erwin, et al.; insecticidal fogging of mostly bare green leaves, some with covering of lichenous or bryophytic plants in terre firme forest, Lot 743 (**USNM**). Paratypes: 1♀: Ecuador: Orellana [labeled Napo in error], Tiputini Biodiversity Station, 216 m, 00°39′10″ S, 76°08′39″ W, 21 October 1998, T.L. Erwin, et al., collectors; Lot # 1932, Transect # T–4 insecticidal fogging of newly bare green leaves, some with of lichenous or bryophytic plants; 1 ♂: Ecuador: Orellana [labeled Napo in error], Tiputini Biodiversity Station, 216 m, 00°39′10″ S, 76°08′39″ W, 4 July 1998, T.L. Erwin, et al., collectors; insecticidal fogging of newly bare green leaves, some with of lichenous or bryophytic plants; Lot # 1876, Transect # T–8 (Figure 21); 1 ♂: Ecuador: Napo, Tiputini Biodiversity Station, 216 m, 00°39′10″ S, 76°08′39″ W, 5 Feb. 1999, T.L. Erwin, et al., collectors; insecticidal fogging of newly bare green leaves, some with covering of lichenous or bryophytic plants; Lot #2085, Transect #T–9; 1 ♀: Ecuador, Orellana Prov. [labeled Napo Prov. in error], Res. Ethnica Waorani, 1 km. S, Onkone Gare Camp, Trans. Ent., 21 June 1994, 220 m, 00°39′10″ S, 76 °26′00″ W, T.L. Erwin, et al., insecticidal fogging of mostly bare green leaves, some with covering of lichenous or bryophytic plants in terre firme forest, At 8 x-trans, 0 m mark Project MAXIMUS Lot 701; 1 ♂: Venezuela, T. F. Amaz. Cerro de la Neblina, Basecamp, 140 m, 00°50′ N, 66°10′ W, 5 February 1985; On low foliage, rainforest trail W. E. Steiner collector (**USNM**); 2 ♂♂: R. Uaupés Taracua; Sv. Amaz. Exp. Roman; 5 april; Swedish Museum of Natural History Stockholm; 1 ♂: the same data; 18 mars (**NHRS**).

#### 3.2.10. *Cylapocoris simplexoides* New Species (Figure 17 and Figure 18)

**Figure 17:**
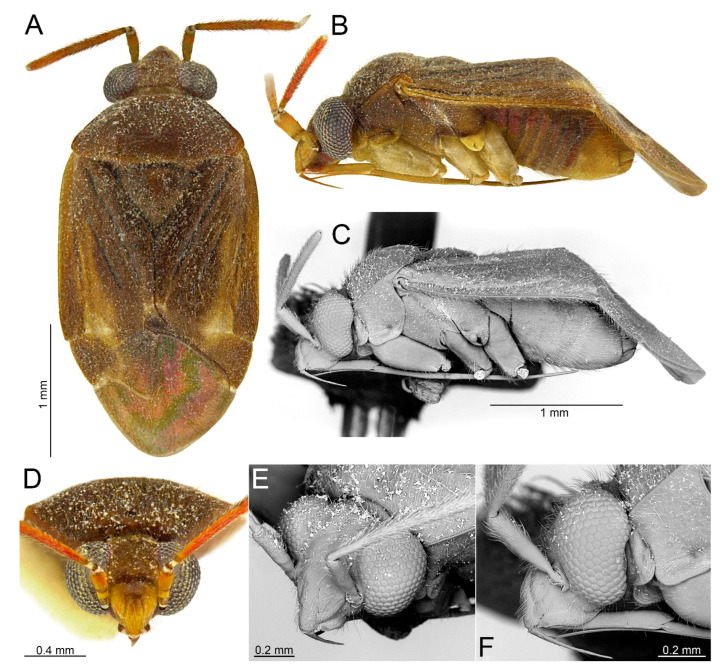
*Cylapocoris simplexoides* sp. nov., holotype: (**A**). Dorsal view; (**B**,**C**). Lateral view; (**D**). Head and pronotum, anterior view; (**E**). Head anterolateral view; (**F**). Head, lateral view.

**Figure 18:**
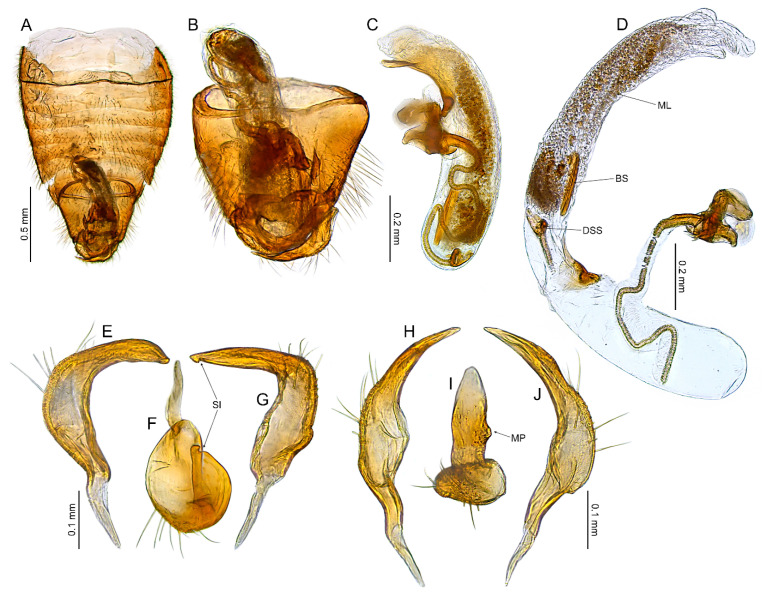
*Cylapocoris simplexoides* sp. nov., holotype: (**A**,**B**). Pygophore: (**A**). Ventral view, (**B**). Dorsal view; (**C**). Aedeagus with theca, left lateral view; (**D**). Aedeagus, theca removed, right lateral view: (**E**–**G**). Left paramere: (**E**). Right lateral view, (**F**). Apical process, dorsal view, (**G**). Left lateral view; (**H**–**J**). Right paramere: (**H**). Right lateral view, (**I**). Apical process, dorsal view, (**J**). Left lateral view. DSS = sclerotized part of ductus seminis inside endosoma; BS = basal sclerite; ML = mesial lobe; MP = medial process; SI = subapical incision.

**Diagnosis**. Recognized by the antennomere II concolor, without any annulation apically (Figure 17A); hemelytron medium brown with small dirty yellow patch on posterior part of endocorium and inner angle of cuneus (Figure 17A); endosoma thin and elongate (Figure 18A–D); sclerotized part of ductus seminis inside endosoma (DSS) short, as long as wide, strongly incrassate, medial lobe (ML) occupying most of endosoma; basal sclerite (BS) present, short, subrectangular (Figure 18C,D).

**Description**. **COLORATION**. Dorsum medium brown with small dirty yellowish areas (Figure 17A). ***Head***. Vertex, frons, ventral half of maxillary plate and buccula medium brown (Figure 17A,B,D); clypeus, mandibular plate and dorsal half of maxillary plate yellow (Figure 17D); antennomere I yellow with medium brown annulation near base and reddish tinge apically (Figure 17B); antennomere II reddish with narrow dark brown annulation basally, broader whitish annulation near base and medium brown annulation bordering the whitish one (Figure 17A,D); antennomeres III and IV missing in the examined specimen; labial segments I and II dirty yellow; segment III medium brown; segment IV dark brown (Figure 17B). ***Thorax***. *Pronotum, mesoscutum, scutellum and hemelytron*. Medium brown (Figure 17A,B); hemelytron with small dirty yellow patch on posterior part of endocorium and inner angle of cuneus; venation surrounding minor cell and outer part of venation surrounding major cell dirty yellow, inner part of major cell venation medium brown (Figure 17A). *Thoracic pleura*. Medium brown; ventral margin of prosternum dirty yellow tinged; metathoracic scent gland evaporative area yellow, except for narrowly brown posterior part (Figure 17B). *Legs*. Coxae yellow; remaining leg segments missing in the examined specimen (Figure 17B). **TEXTURE AND VESTITURE**. Dorsum matte, covered with moderately dense, relatively long semi-recumbent setae (Figure 17A–C,E,F). ***Head***. Weakly rugose, covered with moderately dense, semi-recumbent and erect setae sparser on frons (Figure 17E,F); antennomere I basal half nearly glabrous, with several setae, apical half covered with relatively long, semi-recumbent setae (Figure 17F); antennomere II covered with dense, relatively long, semi–recumbent and erect setae (Figure 17A,C,E). ***Thorax***. Pronotum with sparse and shallow punctation (Figure 17A). *Thoracic pleura*. Covered with sparse, relatively long, semi-recumbent setae (Figure 17B,C). **STRUCTURE**. Body elongate-oval (Figure 17A). ***Head***. Short, portion between apex and clypeus and outer anterior part of eye shorter than eye in lateral view (Figure 17B,F); antennomere II cylindrical (Figure 17A). ***Thorax***. Scutellum weakly convex (Figure 17B,C). **Abdomen**. *Genitalia*. Aedeagus. Endosoma thin and elongate (Figure 18A–D); sclerotized part of ductus seminis inside endosoma (DSS) short, as long as wide, strongly incrassate, medial lobe (ML) occupying most of endosoma; basal sclerite (BS) present, short, subrectangular (Figure 18B–D).

**Measurements**. Holotype ♂: *Body*. Length: 3.14, width: 1.48. *Head*. Length: 0.55, width: 0.87, interocular distance: 0.33. *Antenna*. Length of antennomere I: 0.42, II: 1.01, III: missing, IV: missing. *Labium*. Length of segment I: 0.56, II: 0.58, III: 0.58, IV: 0.50 *Pronotum*. Length: 0.57, width of anterior margin: 0.69, length of lateral margin: 0.54, width of posterior margin: 1.35.

*Female*. Unknown.

**Etymology**. The specific epithet “simplexoides” is used to denote the close phylogenetic affinity to *C. simplex*.

**Biology**. Unknown.

**Distribution**. Peru (Tambopata) (“14” in Figure 22).

**Remarks**. It is most similar to *C. barensis*, *C. simplex*, and *C. fulvus* in sharing the hemelytron with indistinct pale patch on apex of corium and inner angle of cuneus (Figure 17A; Figures 4 and 10 in [25]) and distinctly developed mesial lobe (Figure 18C,D; Figure 5 in [23]; Figures 45 and 85 in [25]). It can be distinguished by straight basal sclerite of endosoma (Figure 18D), which is absent in *C. simplex* (Figure 80 in [25]) and curved, pointed in *C. fulvus* (Figure 45 in [25]).

**Type material**. Holotype ♂: Peru: Tambopata Province, 15 km NE Puerto Maldonado, 18 July 1989, 200 m, J. Ashe, R. Leschen #562 (**KUNHM**).

#### 3.2.11. *Cylapocoris tiquinensis* Carvalho, 1954 (Figure 19F–M, Table 1)

*Cylapocoris tiquiensis* Carvalho 1954 [20]: 508, Pl. I, Figure 5, Pl. 2, Figures l–2, 5 (n. sp.); [55]: 28 (catalog); [21]: 485, 487 (diag., key); [56]: 154 (list); [57]: 22 (catalog); [64]: 7 (list); [2]: 28 (catalog); [25]: 526, Figures 12 and 22 (diagnosis, redescription).

**Diagnosis**. See [25].

**Figure 19:**
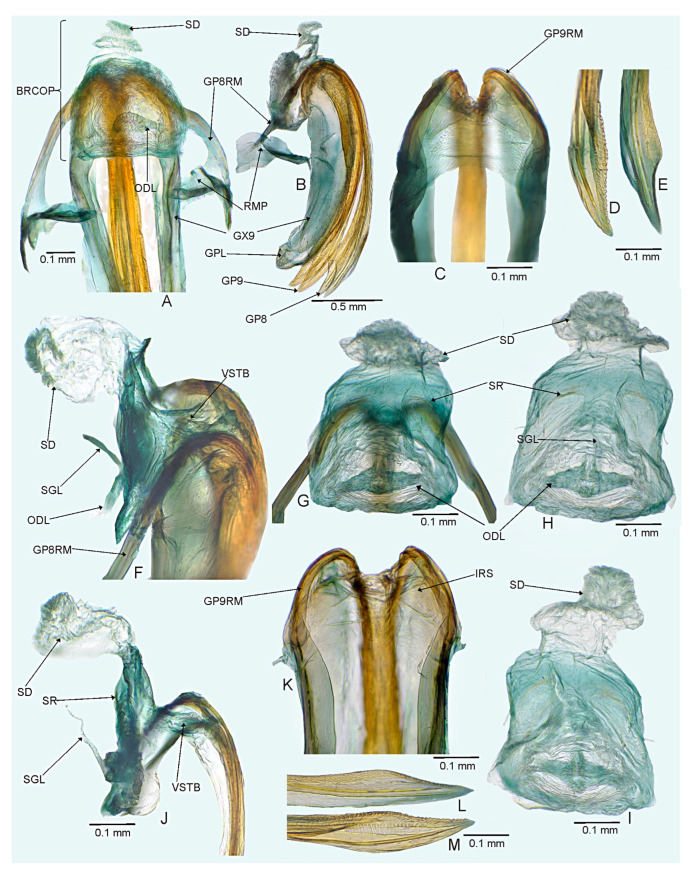
*Cylapocoris simplex* Wolski, 2013, paratype (**A**–**E**) and *C. tiquinensis* Carvalho, 1954, female from Suriname (**F**–**M**) female: (**A**,**B**,**F**). Female genitalia: (**A**). Dorsal view, (**B**). Lateral view, (**F**). Anterolateral view; (**C**,**K**). Bursa copulatrix, posterior wall; (**D**,**E**,**L**,**M**). Ovipositor: (**D**,**L**). Gonapophysis 8, (**E**,**M**). Gonapophysis 9; (**G**,**H**,**J**,**I**). Bursa copulatrix: (**G**,**H**). Dorsal view; (**J**). Lateral view; (**I**). Ventral view. BRCOP = bursa copulatrix; GP8 = gonapophysis 8; GP9 = gonapophysis 9; GP8RM = ramus of gonapophysis 8; GP9RM = ramus of gonapophysis 9; GPL = gonoplac; GX9 = gonocoxae 9; IRS = interramal sclerite; ODL = lateral oviducts; RMP = ramal plate; SD = seminal depository; SGL = spermathecal gland; SR = sclerotized rings; VSTB = vestibulum.

*Female*. *Genitalia*. Bursa copulatrix subrectangular, membranous, with two thin-rimmed sclerotized rings, ellipsoidal, relatively large, situated mediolaterally (Figure 19F–J); posterior wall of bursa copulatrix large, with subcircular interramal sclerite (Figure 19K).

**Distribution**. Brazil (Amazonas), Peru (Tambopata) [20,25], Suriname (this paper) (“10”, “14” and “16” in Figure 22).

**Type material**. Allotype ♀: Amazonas, Brasil, Taracua, 6–49, JCM Carvalho col; Allotype [reddish label]; Carvalho to Drake Coll. 1993 (**USNM**).

**Additional examined specimens**. 1♀: Peru: Tambopata Reserve, 30 km SW Puerto Maldonado, IX/19-X/10/84; 12°12′ S, 69°16′ W Trop[ical] Moist F[o]r[e]st., D.A. Grimaldi; reared from fungus: Auricularia (**AMNH**); 1 ♀: Tiquié, Amazonas, JCM Carvalho Col. 1949 (**USNM**); 1 ♀: Suriname: Commewinje Akintoosoela, Celos Camp, 39 km SE Suriname River bridge, road to Redi Doti, 40 m, 5°16′17″ N, 54°55′15″ W, 30 June 1999; Z.H. Falin, SUR1F99 128 EX: fogging woody orange bracket fungus (**KUNHM**).

#### 3.2.12. *Cylapocoris vittatus* Wolski, 2017 (Figure 20, Table 1)

*Cylapocoris vittatus* Wolski, 2017 [27]: 288, Figures 8 and 9 (n. sp.)

**Diagnosis**. See [27].

**Figure 20:**
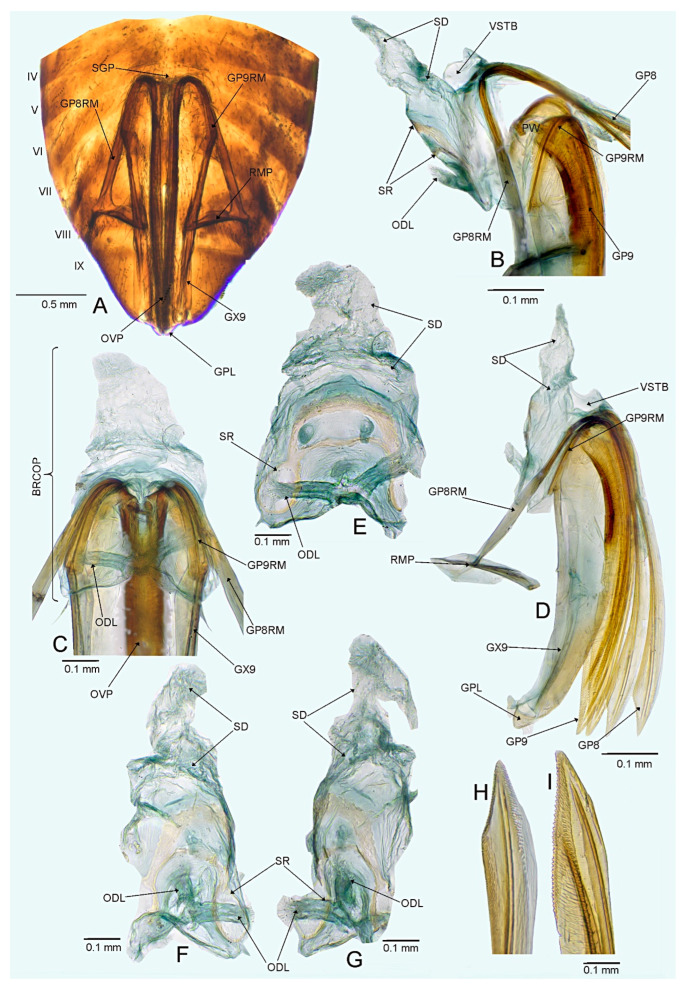
*Cylapocoris vittatus* Wolski, 2017, female from Ecuador: (**A**). Abdomen, ventral view; (**B**–**D**). Female genitalia: (**B**,**D**). Lateral view, (**C**). Dorsal view; (**E**–**G**). Bursa copulatrix: **E**. Dorsal view, (**F**). Ventrolateral view, (**G**). Dorsolateral view; (**H**,**I**). Ovipositor: (**H**). Gonapophysis 8, (**I**). Gonapophysis 9. BRCOP = bursa copulatrix; GP8 = gonapophysis 8; GP9 = gonapophysis 9; GP8RM = ramus of gonapophysis 8; GP9RM = ramus of gonapophysis 9; GPL = gonoplac; GX9 = gonocoxae 9; ODL = lateral oviducts; OVP = ovipositor; PW = posterior wall of bursa copulatrix; RMP = ramal plate; SD = seminal depository; SGL = spermathecal gland; SGP = sub-genital plate; SR = sclerotized rings; VSTB = vestibulum.

*Female*. *Genitalia*. Bursa copulatrix semicircular, dorsal wall of bursa copulatrix with broad subsemicircular thick-rimmed area occupying anterior half; sclerotized rings situated posterolaterally, thick-rimmed, inner portion thicker, fused with anterior sclerotization (Figure 20E–G).

**Distribution**. Costa Rica (Cartago Province), Ecuador (Santo Domingo de los Tsáchilas Province), Panama (Colón Province) (Wolski 2017, this paper) (“4”, “5” and “13” in Figure 22).

**Type material**. Holotype ♀: Costa Rica, Cartago Province, Pejibaye, 22–24 March 1987, W. E. Steiner (**USNM**); paratypes: 1♀: Canal Zone: Barro Colorado, 13–VII 1924, N. Banks; 1 female: Santo Domingo de Colorados, Ecuador, 6 March 1973, M. A. Deyrup; 1♀: Santo Domingo de Colorados, Ecuador, 8 February 1973, M. A. Deyrup (**AMNH**).

**Additional examined material**. 1♀: Ecuador: Pichincha Maquipucuna Forest Reserve 50 km NW Quito, 1720 m, 21 December 1991, C. Carlton, R. Leschen #38, ex: flat ascomycete; KU Loan 2017 (**KUNHM**).

#### 3.2.13. Genus: *Cylapocoroides* Carvalho, 1989 [65]

*Cylapocoroides* Carvalho 1989 [65]: 444 (gen. nov.); *Cylapocoroides*: [59]: 482 (list); [57]: 22, [58] (online catalogue); [17]: 49 (catalogue). Type species: *Cylapocoroides centralis* Carvalho, 1989 (original designation)

**Figure 21:**
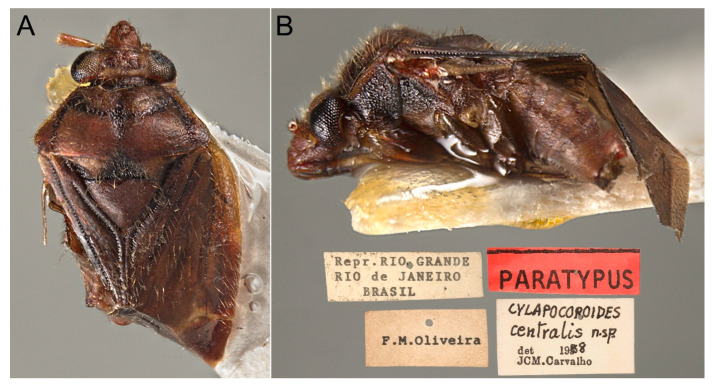
*Cylapocoroides centralis* Carvalho, 1989, paratype: (**A**). Dorsal view; (**B**). Lateral view.

**Diagnosis**. Recognized by the following set of features: body ovoid (Figure 21A); frons weakly sloping forward (Figure 21B); vertex with weakly developed carina (Figure 21A); scent gland evaporative area relatively broad, triangular (Figure 21B); suture between pronotal collar and remainder of pronotum scalloped (Figure 21A); calli broad, weakly upraised, delimited by broad punctate longitudinal furrow extended to middle of posterior lobe, calli delimited from the remainder of pronotum by broad punctate transverse furrow reaching lateral margins, pronotum also with two broad punctate longitudinal furrows each situated sublaterally, from anterior to humeral angle (Figure 21A); corium and clavus with a row of punctures along R + M, anal veins and claval commissure; exocorium (embolium) broad (Figure 21A).

**Redescription** (based on [24] and paratype from Rio de Janeiro). *Male*. Macropterous. **TEXTURE AND VESTITURE**. Dorsum covered with dense, erect, relatively long setae (Figure 21A,B). ***Head***. Covered with dense, long, erect setae, mostly rugose; vertex with depressed punctate area medially; antennomere I covered with relatively long, reclining setae (Figure 21A), remaining antennomeres missing in the examined specimen. ***Thorax***. *Pronotum*. Weakly shining; suture between pronotal collar and remainder of pronotum scalloped; pronotum with depressed punctate areas (see below for structure and arrangement of these areas). *Mesoscutum and scutellum*. Smooth, depressed area between mesoscutum and scutellum punctate. *Thoracic pleura*. Covered with sparse, dense, long setae; propleuron rugopunctate; remaining pleura rugose. *Hemelytron*. Weakly shining; corium and clavus with a row of punctures along R + M, anal veins and claval commissure; membrane with setae on surface outside cells. **STRUCTURE**. Macropterous. Body ovoid, stout (Figure 21A). ***Head***. Frons weakly sloping forward, longer than wide in anterior view, about as high as long in lateral view; vertex somewhat carinate posteriorly (Figure 21A); clypeus not separated from frons, clypeal base situated above ventral margin of eye; antennal insertion contiguous with sulcus between maxillary and mandibular plates (Figure 21B); eyes contiguous with pronotal collar; eye relatively large, reniform, its ventral margin reaching gula (Figure 21B); mandibular plate without sulcus posteriorly; labium thin and long, reaching pygophore [24]; segment I reaching xyphus, subdivided near medial part (Figure 21B); ***Thorax***. *Pronotum*. Collar distinct, thin, depressed, separated from remainder of pronotum by deep suture (Figure 21A,B); calli broad and short, moderately convex, delimited by broad longitudinal furrow extended to middle of posterior lobe, calli delimited from the remainder of pronotum by broad transverse furrow reaching lateral margins, pronotum also with two broad punctate longitudinal furrows each situated sublaterally, from anterior to humeral angle (Figure 21A); lateral margin weakly elevated, strongly carinate, straight; posterior margin weakly convex medially (Figure 21A). *Mesoscutum and scutellum*. Mesoscutum well exposed; scutellum weakly convex (Figure 21A,B). *Thoracic pleura*. Metathoracic spiracle slit-like without microsculpture; scent gland evaporative area relatively broad, triangular; ostiolar peritreme weakly upraised, round (Figure 21B). *Hemelytron*. Claval commissure about as long as scutellum; costal fracture present, cuneus well developed as wide as long; exocorium (embolium) wide; membrane with two cells, major cell large, nearly rectangular [24]. ***Abdomen***. *Genitalia*. As described and depicted by Carvalho ([24]: Figures 7–9).

#### 3.2.14. *Cylapocoroides centralis* Carvalho, 1989

*Cylapocoroides centralis* Carvalho 1989 [24]: 446, Figures 6–9 (n. sp.)

*Cylapocoroides centralis*: [59]: 490 (list); [57]: 22 (catalogue), [58] (online catalog); [2]: 29 (catalogue)

**Diagnosis**. See the generic diagnosis.

**Redescription** (based on [24] and paratype from Rio de Janeiro)**. COLORATION**. Dorsum dark castaneous. ***Head***. Dark castaneous; antenna castaneous, becoming darker toward apex and paler near base; vertex weakly tinged with castaneous; labium yellowish dark castaneous. ***Thorax***. *Pronotum*. From dark castaneous with black elongated depressed areas castaneous to black with brown reddish patches near calli, in the middle of disc, and humeral angles. *Mesoscutum and scutellum*. Mesoscutum black with paler patches; scutellum dark castaneous. *Thoracic pleura*. From dark castaneous to nearly black; evaporative areas paler. *Hemelytron*. From castaneous, paler on exocorium to almost entirely dark castaneous, area of corium bordering cuneus and base of cuneus narrowly paler; membrane fuscous, venation with transversal part paler *Legs*. Coxae from entirely dark castaneous to dark castaneous with paler apex and base. ***Abdomen***. Dark castaneous.

**Measurements**. Holotype ♂ (from Carvalho 1989): *Body*. Length: 4.0, width 2.1. *Head*. Length: 0.3, width: 1.0, vertex width: 0.44. *Antenna*. Length of antennomere: I: 0.3, II: 0.9, III: 0.4, IV (missing). *Pronotum*. Length: 0.6, width of posterior margin: 1.6. *Cuneus*. Length: 0.58, width 0.44.

**Biology**. Unknown.

**Distribution**. Brazil (Rio de Janeiro, São Paulo).

**Type material**. Holotype ♂: Rio de Janeiro, Floresta da Tijuca, Brasil, GB (Estado da Guanabara, atual Estado do Rio de Janeiro), IV.1966, Alvarenga col. (Museu Nacional, Rio de Janeiro (**MNRJ**, not examined). Paratype: ♂: Represa Rio Grande, Rio de Janeiro, Brasil, F.M. Oliveira (**USP**). Paratype: ♂: Estarto Biológica de Boraceia, Salesópolis, SP. (Sao Paulo), Brasil,13.IX.1960, K. Lenko (**USP**, not examined).

**Figure 22:**
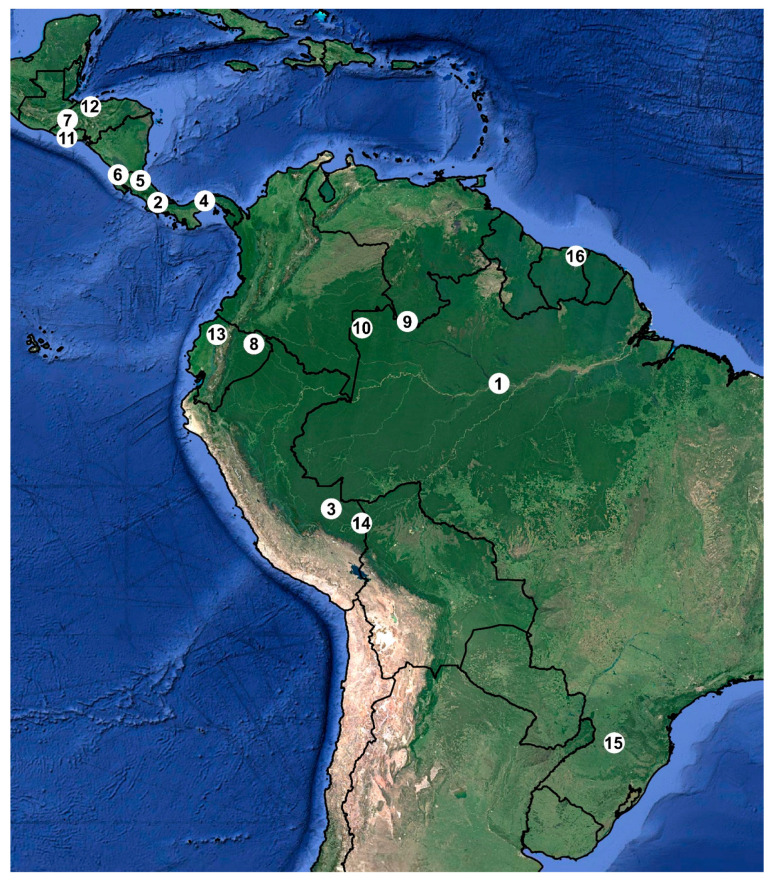
Distribution map of *Cylapocoris* spp. *C. barensis* (1), *C. bimaculatus* (2), *C. brailovskyi* (2), *C. brooksi* (3), *C. carvalhoi* (2), *C. castaneus* (4), *C. costaricaensis* (5), *C. cucullatus* (5–7), *C. fulvus* (8), *C. funebris* (2, 6), *C. laevigatus* (9), *C. marmoreus* (5), *C. pilosus* (10), *C. plectipennis* (6), *C. salvadorensis* (11, 12), *C. scutellatus* (13), *C. simplex* (8–10), *C. simplexoides* (14), *C. sulinus* (15), *C. tiquiensis* (10, 14, 16), *C. vittatus* (4, 5, 13).

**Key to species of *Cylapocoris***
  1. Pronotum devoid of punctation (Figures 1, 6–7 and 38 in [25])…………………………**2**
  - Pronotum with more or less developed punctation (Figure 3D and Figure 9A)……………**4**
  2. Hemelytra dark yellow tinged with dark brown and brown and with yellow stripes along medial fracture and R + M and claval veins (Figure 3 in [26])……………………………………………………***Cylapocoris vittatus* Wolski, 2017**
  - Hemelytron entirely castaneous or dark castaneous (Figures 1, 6 and 7 in [25])…………………………………………………………………………………………**3**
  3. Body length less than 4.0 mm; head, when viewed laterally, with gula and posterior half of buccula only slightly differing from remainder of head in coloration (Figure 13 in [25]); endosoma with right lateral margin of DSS straight (Figure 23 in [25]); left paramere distinctly curved, with apical process, when viewed dorsally, with left lateral margin distinctly convex medially (Figures 26 and 27 in [25])…………………………………………………………***C. castaneus* (Carvalho, 1989)**
  - Body length more than 4.0 mm; head in lateral view mostly dark castaneous, with contrastingly yellow gula and posterior half of buccula (Figure 18 in [25]); endosoma with right lateral margin of DSS convex medially (Figure 51 in [25]); left paramere only weakly curved, with apical process, when viewed dorsally, with left lateral margin moderately rounded, not distinctly curved medially (Figures 52 and 53 in [25])……………………………………………………………***C. laevigatus* Wolski, 2013**
  4. Claval vein yellow along entire length (Figure 3A and Figure 12B,E)…………………………**5**
  - Clavus usually unicolored, without yellow stripe along entire length……………………**6**
  5. Posterior lobe of pronotum mostly dark brown with two, relatively large yellow patches situated sublaterally (Figure 3A)…………………………***C. bimaculatus* n. sp.**
  - Posterior lobe of pronotum yellow, except for lateral portion (Figure 12B,E)………………………………………………………***C. salvadorensis* Carvalho, 1989**
  6. Basal half of antennomere II dark yellow (Figure 12 in [25]) ………………………………………………………………***C. tiquinesis* Carvalho, 1954**
  - Antennomere II from entirely yellow to dark brown (e.g., Figure 6A, Figure 15A and Figure 17A) sometimes with pale annulation apically (e.g., Figure 3A, Figure 9A and Figure 12E)…………………………………………………………………………………………**7**
  7. Hemelytra uniformly dark castaneous to almost black, without any pale patch on apex of corium (e.g., Figure 6A and Figure 9A); DSS comparatively long, longer than wide (e.g., Figure 4C, Figure 7C and Figure 10C); ML strongly reduced or absent (e.g., Figure 4C, Figure 7C and Figure 10C); at least lateral sclerite well developed (e.g., Figure 4C, Figure 7C and Figure 10C)………………………………………………………………………………………….**8**
  - Hemelytra brown to dark brown with apical portion of endocorium and inner angle of cuneus with yellowish patch (e.g., Figure 17A; Figures 4, 10 and 11 in [25]) or with numerous dirty yellowish or stramineous patches on clavus, corium, and extreme apex of cuneus (e.g., Figure 15A; Figure 8 in [25]); DSS strongly shortened, as long as wide (e.g., Figure 16D and Figure 18D); ML distinctly developed, occupying most of endosoma (e.g., Figure 16D and Figure 18D); medial (MS) and lateral (LS) sclerites always absent (e.g., Figure 16D and Figure 18D)…………………………………………………………………………………………**15**
  8. Antennomere II uniformly colored, without pale annulation apically (Figure 6A)…**9**
  - Antennomere II with contrastingly pale, yellowish annulation apically (Figure 3A, Figure 9A and Figure 12E)………………………………………………………………………………**10**
  9. Pronotum mostly ochraceous, calli and lateral margins dark brown (Figure 6A); endosomal apical sclerite absent (Figure 7C,D); medial sclerite small, plate-like (Figure 7C,D)………………………………………………………………***C. brooksi* n. sp.**
  - Pronotum dark brown with paler, dirty yellowish, narrow tinge along entire posterior margin; apical sclerite present (Figure 62 in [25]); medial sclerite well developed, tapering toward apex, sharply pointed (Figure 62 in [25])……………………………………………………………***C. pilosus* Carvalho, 1954**
  10. Labrum somewhat compressed laterally, protruding forward; membrane covered with very dense, minute setae (Figure 90 in [25]); lateral endosomal sclerite large occupying most of endosoma (Figure 28 in [25]); apical sclerite absent (Figure 28 in [25])………………………………………………………***C. costaricaensis* Wolski, 2013**
  - Labrum not compressed laterally, not protruding forward; membrane devoid of setae or covered with a few setae situated near lateral margin (as on Figure 91 in [25]); endosomal lateral sclerite (LS) much smaller, not occupying most of endosoma (Figures 40, 62, 66 in [25]); apical sclerite (AS) present (Figures 40, 62 and 66 in [25])…………………………………………………………………………………………**11**
  11. Pronotal collar entirely dark brown, without yellowish tinge medially (Figure 9 in [25])…………………………………………………………***C. plectipennis* Wolski, 2013**
  - Pronotal collar with yellowish patch medially (Figure 32 in [25], Figure 1 in [26])…………………………………………………………………………………………**12**
  12. Pronotum dark yellow, broadly tinged with dark brown anteriorly and with three indistinct brown stripes on posterior lobe (Figure 1 in [26])………………………………………………………………***C. brailovskyi* Wolski, 2017**
  - Pronotum entirely dark brown to blackish (Figure 9A)………………………………**13**
  13. Body length less than 3.5 mm…………………………………………………………….**14**
  - Body length more than 3.5 mm………………………………***C. funebris* (Distant, 1883)**
  14. Endosoma with mesal sclerite plate-like, apical sclerite tapering, beak-shaped (Figure 10C,D)……………………………………………………………………***C. carvalhoi* n. sp.**
  - Endosoma with mesal sclerite elongated, pointed, apical sclerite sub-ellipsoid (Figure 40 in [25])………………………………………………………***C. cucullatus* Wolski, 2013**
  15. Hemelytra with mottled coloration (Figure 15A; Figure 8 in [25])……………………**16**
  - Hemelytra brown to dark brown, except for small patch on apex of endocorium and inner angle of cuneus (e.g., Figure 17A; Figures 4 and 10 in [25])……………………**17**
  16. Scutellum with broad, longitudinal, stramineous reddish framed stripe medially (Figure 15A)……………………………………………………………***C. scutellatus* n. sp.**
  - Scutellum without broad, longitudinal stripe, only with yellowish patch basally (Figure 8 in [25])……………………………………………***C. marmoreus* Wolski, 2013**
  17. Pronotum yellow with brownish calli area and with two distinct, brown, triangular patches medially of posterior lobe, each bordering posterior margin (Figure 11 in [25])………………………………………………***C. sulinus* Carvalho and Gomes, 1971**
  - Pronotum mostly dark castaneous to dark black, without any triangular patches bordering posterior margin, sometimes with yellowish, relatively broad stripe along posterior margin (Figures 4 and 10 in [25])……………………………………………**18**
  18. Pronotal posterior lobe paler than remainder pronotum (Figure 4 in [25]) ………………………………………………………………………***C. fulvus* Wolski, 2013**
  - Pronotum entirely medium brown to dark brown (Figure 17A; Figure 10 in [25])…………………………………………………………………………………………**19**
  19. Body length less than 4.0 mm……………………………………………………………**20**
  - Body length more than 4.0 mm………………………………***C. barensis* Carvalho, 1982**
  20. Endosoma with short, straight sclerite basally (Figure 18D)……***C. simplexoides* n. sp.**
  - Endosoma without any sclerite (Figure 80 in [25])……………***C. simplex* Wolski, 2013**

## 4. Discussion

This study was partly aimed to test the monophyly of *Cylapocoris* and shed light on the relationships among its species, as well as determine the phylogenetic position of the genus based on the morphological data. Our findings support previous hypotheses regarding the position of *Cylapocoris* and the relationships between its species, as established in the taxonomic studies [25,26]. The current analysis confirms this author’s suggestion of a possible affinity between *Cylapocoris* and *Cylapocoroides* and provides compelling evidence for the sister-group relationship between these taxa, forming a monophyletic group with strong support values (97%). This conclusion is supported by multiple morphological characters. Additionally, *Cylapocoris* is confirmed as a monophyletic group with high support values (95%). Our analysis also supports the observation made by Wolski [25] that the genus *Adcylapocoris* Carvalho, 1989 should be considered a junior synonym of *Cylapocoris*, as we have found strong evidence by nesting *C. castaneus* deeply within *Cylapocoris*. Furthermore, our analysis reveals two main subgroupings within *Cylapocoris*: the *simplex* clade (node 15), with no statistical support and the *pilosus* clade (node 22), with weak nodal support. The assemblages observed within the *simplex* clade are statistically supported, although the strength of support varies from weak to moderate. The groupings identified within the *pilosus* clade lack statistical support. Additionally, within this clade, approximately half of the species exhibit an unresolved polytomy in the EW analysis (clade 23). These findings underscore the necessity for additional evaluation of the phylogenetic relationships within the genus, which could potentially be achieved by incorporating multiple datasets (see below).

Although not the primary focus of this study, our analysis reveals the presence of certain generic groups suggested in previous studies and provides a glimpse into the relationships at deeper nodes, i.e., the tribes of Cylapinae. The present study strongly supports the monophyly of the Neotropical group of genera belonging to the tribe Cylapini, the *Cylapus* complex (clade 3), as established by Wolski [9] in a morphology-based phylogenetic study. Moreover, our analysis provides robust statistical support (100%) for the grouping composed of *Comefulvius*, *Incafulvius*, *Henryfulvius*, and *Xenocylapus* (*Xenocylapus* complex, clade 10), which was previously identified by Chérot et al. [46] and Wolski [35] in their taxonomic studies. This assemblage is decisively supported by a number of character states.

Furthermore, our analysis reveals two major groupings within the subfamily. The first comprises Bothriomirini and Cylapini (clade 1), receiving strong statistical support. This phylogeny confirms the monophyly of Bothriomirini as showed in the analyses based solely on morphology [4,6,9,14] and combined, molecular and morphological datasets [5,14]. Our analyses align with previous morphology-based analyses [4,6,9,14] confirming the monophyly of the assemblage composed of Cylapini and Bothriomirini and establishing their sister-group relationship. These topologies deviated from the topology proposed by Namyatova and Cassis [5] in their total-evidence analysis, as they revealed an unresolved position of the Bothriomirini.

The second major grouping on our phylogenetic tree includes Psallopini, Rhinomirini, and Fulviini (clade 4), and is strongly supported (100% SRS). It is characterized by several distinctive character states. The presence of the grouping including Psallopini, Rhinomirini, and Fulviini is consistent with the results of previous phylogenetic analyses based solely on morphological data conducted by Namyatova and Cassis [6], Wolski [9], and Tyts et al. [14], as well as the total evidence analysis performed by Tyts et al. [14]. In all these analyses, the position of *Psallops* varied from being placed as a sister group to the remaining taxa within this clade (this analysis and [9]) to being nested more deeply within Fulviini [6,14]. This evidence supports the conclusion made by Wolski and Henry [66] that Psallopinae should be considered within the subfamily Cylapinae. On the other hand, Namyatova and Cassis [5] showed *Psallops* as a sister group to cylapines in the total evidence analysis. Our present analysis showed the clade including Rhinomirini and Fulviini which is in line with the results of the other morphology-based analyses [6,9] and contrasts with total-evidence approaches either suggesting the phylogenetic proximity of Rhinomirini with Cylapini and Vanniini [5] or that Rhinomirini is nested in a separate clade with *Psallops* [14]. Wolski [9] and Tyts et al. [14] suggested that this incongruence between the tree topologies obtained using different datasets may be caused by the widespread convergent evolution and many characters used in different morphological analyses may be affected by homoplasy, reducing their phylogenetic significance. Tyts et al. [14] showed that total-evidence analysis gives strong supports for generic complexes and suggested that molecular and morphological approaches may have a complementary role in creating more stable classification. Given the low supports for most of the internal nodes within *Cylapocoris* and most of the nodes within the clade Rhinomirini + Fulviini, it is likely that to receive the more stable phylogeny within the genus and at the deeper nodes, the inclusion of the combined datasets is required. Our study represents the most densely sampled comparative approach ever performed for the genus *Cylapocoris*. The robust morphological dataset here presented is important for future studies integrating multiple datasets aiming at resolving the phylogeny of the genus and intertribal relationships.

## 5. Conclusions

Our research presents a significant contribution to the understanding of taxonomy and phylogeny within of the genus *Cylapocoris*. By extensively analyzing morphological data, including the novel study of female genitalia, we were able to offer an updated diagnosis and description of the genus. Our study represents the first effort to test the monophyly of *Cylapocoris* and shed light on the relationships among its species, as well as determine the phylogenetic position of the genus. We confirm the monophyly of *Cylapocoris* and its sister-group relationship with *Cylapocoroides*. Additionally, we identify subgroupings within *Cylapocoris*. We also support the monophyly of the *Cylapus* and *Xenocylapus* complexes. Our findings also highlight the need for integrating molecular and morphological approaches to achieve a more stable classification within the Cylapinae what was suggested by the previous authors. Further research utilizing combined datasets is necessary to obtain a more reliable phylogeny at both the genus and deeper node levels. Our study lays a strong foundation for future investigations into the phylogeny of the genus and intertribal relationships by offering comprehensive and reliable morphological information.

## Data Availability

Data are contained within the article or Supplementary Materials.

## Supplementary Materials

The following supporting information can be downloaded at: https://www.mdpi.com/article/10.3390/insects14090721/s1, File S1: The morphological dataset used for the phylogenetic analysis.

## References

[1] Kirkaldy G.W. (1903). Einige neue und wenig bekannte Rhynchoten. Einege neue und wenig bekannte Rhynchoten. Wien. Entomol. Ztg..

[2] Gorczyca J. (2006). The Catalogue of Subfamily Cylapinae Kirkaldy, 1903 of the World (Hemiptera, Heteroptera, Miridae).

[3] Konstantinov F.V. (2012). A New Species of *Palaucoris* (Heteroptera: Miridae) from Sulawesi. Entomol. Am..

[4] Namyatova A.A., Contos P., Cassis G. (2018). New species, taxonomy, phylogeny, and distribution of the tropical tribe Bothriomirini (Insecta: Heteroptera: Miridae: Cylapinae). Insect Syst. Evol..

[5] Namyatova A.A., Cassis G. (2019). Total-evidence phylogeny of the Rhinomirini, taxonomic review of its subgroupings (Insecta: Heteroptera: Miridae: Cylapinae) and description of new Australian taxa. Zool. J. Linn. Soc..

[6] Namyatova A.A., Cassis G. (2019). First record of the subfamily Psallopinae (Heteroptera: Miridae) from Australia and discussion of its systematic position and diagnostic characters. Aust. Entomol..

[7] Namyatova A.A., Cassis G. (2021). Five new genera of the subfamily Cylapinae (Insecta, Heteroptera, Miridae) from Australia. Zookeys.

[8] Namyatova A.A., Cassis G. (2022). Review of Australian Cylapinae (Hemiptera: Heteroptera: Miridae) with key to genera and descriptions of new species. Insect Syst. Evol..

[9] Wolski A. (2021). Revised classification of the New World Cylapini (Heteroptera: Miridae: Cylapinae): Taxonomic review of the genera *Cylapinus*, *Cylapoides* and *Peltidocylapus* and a morphology-based phylogenetic analysis of Cylapini. Zootaxa (Monogr.).

[10] Schuh R.T., Weirauch C. (2020). True Bugs of the World (Hemiptera: Heteroptera): Classification and Natural History.

[11] Namyatova A.A., Konstantinov F.V., Cassis G. (2016). Phylogeny and systematics of the subfamily Bryocorinae based on morphology with emphasis on the tribe Dicyphini sensu Schuh. Syst. Entomol..

[12] Cassis G., Schwartz M., Moulds T. (2003). Systematics and New Taxa of the Vannius Complex (Hemiptera: Miridae: Cylapinae) from the Australian Region. Mem. Qld. Mus..

[13] Cassis G., Monteith G.B. (2006). A new genus and species of Cylapinae from New Caledonia with re-analysis of the *Vannius* complex phylogeny (Heteroptera: Miridae). Mem. Qld. Mus..

[14] Tyts V.D., Namyatova A.A., Konstantinov F.V. (2022). Phylogeny of the *Rhinocylapus* complex (Heteroptera, Miridae, Cylapinae, Fulviini). Invertebr. Syst..

[15] Cassis G., Schuh R.T. (2011). Systematics, biodiversity, biogeography, and host associations of the Miridae (Insecta: Hemiptera: Heteroptera: Cimicomorpha). Annu. Rev. Entomol..

[16] Gossner M.M., Damken C., Ulyshen M. (2018). Diversity and ecology of saproxylic Hemiptera. Saproxylic Insects.

[17] Gorczyca J. (2000). A Systematic Studies of Cylapinae with a Revision of the Afrotropical Region (Heteroptera, Miridae).

[18] Wheeler Q.D., Wheeler A.G. (1994). Mycophagous Miridae? Associations of Cylapinae (Heteroptera) with pyrenomycete fungi (Euascomycetes: Xylariaceae). J. N. Y. Entomol. Soc..

[19] Wolski A., Yasunaga T. (2016). Taxonomic review of the fungal-inhabiting plant bug genera Bothriomiris and Dashymenia (Hemiptera: Heteroptera: Miridae: Cylapinae: Bothriomirini), with descriptions of two new species of *Dashymenia* from Thailand. Raffles Bull. Zool..

[20] Carvalho J.C.M. (1954). Neotropical Miridae, 74: Two new genera of Cylapinae from Brazil (Hemiptera). Proc. Iowa Acad. Sci..

[21] Carvalho J.C.M., Gomes I.P. (1971). Mirideos neotropicais, CXXXVI: Genero Cylapocoris Carvalho com a descriçao de uma nova especie (Hemipera). Rev. Bras. Biol..

[22] Carvalho J.C.M. (1976). Analecta Miridologica: Concerning changes of taxonomic positions of some genera and species (Hemiptera). Rev. Bras. Biol..

[23] Carvalho J.C.M. (1982). Mirideos neotropicais CCXXXIX: Descriçoes de algumas espécies de Cylapinae do Amazonas (Hemiptera). Acta Amaz..

[24] Carvalho J.C.M. (1989). Mirideos neotropicais, CCCVI: Novos generos e espécies da tribo Cylapini Kirkaldy (Hemiptera). Bol. Mus. Para. Emilio Goeldi Cienc. Nat. Zool..

[25] Wolski A. (2013). Revision of the plant bug genus *Cylapocoris* (Hemiptera: Heteroptera: Miridae: Cylapinae), with descriptions of seven new species from Costa Rica, Brazil, Ecuador, and Venezuela. Zootaxa.

[26] Wolski A. (2017). Taxonomic review of the plant bug genera *Amapacylapus* and *Cylapus* with descriptions of two new species and a key to the genera of Cylapini (Hemiptera: Heteroptera: Miridae). Acta Entomol. Mus. Natl. Pragae.

[27] Herrich-Schaeffer G.A.W. (1835). Nomenclatur Entomologicus. Verzeichniss der Europäischen Insecten.

[28] Schuh R.T. (1976). Pretarsal structure in the Miridae (Hemiptera) with a cladistic analysis of relationships within the family. Am. Mus. Novit..

[29] Schuh R.T., Weirauch C., Wheeler W.C. (2009). Phylogenetic relationships within the Cimicomorpha (Hemiptera: Heteroptera): A total-evidence analysis. Syst. Entomol..

[30] Fitch W.M. (1971). Toward defining the course of evolution: Minimum change for a specific tree topology. Syst. Entomol..

[31] Wilkinson M. (1992). Ordered versus unordered characters. Cladistics.

[32] Maddison W.P., Maddison D.R. (2019). Mesquite: A Modular System for Evolutionary Analysis. Version 3.61. http://www.mesquiteproject.org.

[33] Malipatil M.B., Masłowski A., Dobosz R., Taszakowski A. (2022). *Meschia brevirostris* sp. nov., from New Caledonia (Hemiptera: Heteroptera: Lygaeoidea: Meschiidae). Zootaxa.

[34] Pluot-Sigwalt D., Matocq A. (2017). An investigation of the roof of the genital chamber in female plant-bugs with special emphasis on the “dorsal sac” (Hemiptera: Heteroptera: Miridae). Ann. Société Entomol. Fr..

[35] Wolski A. (2014). Revision of the plant bug genus *Xenocylapus* Bergroth (Hemiptera: Heteroptera: Miridae: Cylapinae), with a description of *Henryfulvius gracilis*—A new cylapine genus and species from Ecuador. Ann. Soc. Entomol. Fr..

[36] Davis N.T. (1955). Morphology of the female organs of reproduction in the Miridae (Hemiptera). Ann. Entomol. Soc. Am..

[37] Taszakowski A., Kim J., Masłowski A., Herczek A., Jung S. (2022). *Kohnometopus yasunagai* sp. nov., (Hemiptera, Heteroptera, Miridae, Isometopinae) from Peninsular Malaysia. Zootaxa.

[38] Kerzhner I.M., Konstantinov F.V. (1999). Structure of the aedeagus in Miridae (Heteroptera) and its bearing to suprageneric classification. Acta Soc. Zool. Bohem..

[39] Konstantinov F.V. (2003). Male genitalia in Miridae (Heteroptera) and their significance for suprageneric classification of the family. Part I: General review, Isometopinae and Psallopinae. Belg. J. Entomol..

[40] Goloboff P.A., Farris J.S., Nixon K.C. (2008). TNT, a free program for phylogenetic analysis. Cladistics.

[41] Goloboff P.A. (1993). Estimating character weights during tree search. Cladistics.

[42] Nixon K.C. (2002). Winclada.

[43] Bremer K. (1994). Branch support and tree stability. Cladistics.

[44] Goloboff P.A., Farris J.S., Källersjö M., Oxelman B., Ramírez M., Szumik C.A. (2003). Improvements to resampling measures of group support. Cladistics.

[45] Wolski A., Gorczyca J. (2014). Notes on the genera *Peritropisca* Carvalho & Lorenzato and *Rewafulvius* Carvalho (Hemiptera: Heteroptera: Miridae: Cylapinae), with the description of a new species of *Peritropisca* from Indonesia. Zootaxa.

[46] Chérot F., Carpintero D.L., Wolski A. (2014). New record and redescription of the monotypic genus Comefulvius Carvalho & Carpintero, 1985 (Hemiptera: Heteroptera: Miridae: Cylapinae). Zootaxa.

[47] van Doesburg P.H. (1985). A new species of *Xenocylapus* Bergroth, 1922, from Surinam (Heteroptera, Miridae, Cylapinae). Entomolog. Ber..

[48] Wolski A., Gorczyca J. (2012). Plant bugs of the tribe Bothriomirini (Hemiptera: Heteroptera: Miridae: Cylapinae) from the Oriental Region: Descriptions of eight new species and keys to Oriental genera and species of *Bothriomiris* Kirkaldy, *Dashymenia* Poppius, and *Dashymeniella* Poppius. Zootaxa.

[49] Wolski A., Gorczyca J., Yasunaga T. (2018). Taxonomic review of the *bifenestratus* species group of the genus *Fulvius* Stål with descriptions of two new species (Hemiptera, Heteroptera, Miridae, Cylapinae). Zookeys.

[50] Namyatova A., Cassis G. (2016). Revision of the staphylinoid and ground-dwelling genus *Carvalhoma* Slater & Gross (Insecta: Heteroptera: Miridae: Cylapinae) of Australia. Eur. J. Taxon..

[51] Gorczyca J., Wolski A., Taszakowski A. (2020). The first record of the genus *Fulvius* Stål, 1862 (Heteroptera: Miridae: Cylapinae) from continental China with description of a new species. Bonn Zool. Bull..

[52] SAGA Development Team (2023). System for Automated Geoscientific Analyses (SAGA).

[53] Uhler P.R. (1886). Check-List of the Hemiptera Heteroptera of North America.

[54] Carvalho J.C.M. (1955). Keys to the genera of Miridae of the world (Hemiptera). Bol. Mus. Para. Emilio Goeldi Cienc. Nat. Zool..

[55] Carvalho J.C.M. (1957). Catalogue of the Miridae of the World. Part I. Subfamilies Cylapinae, Deraeocorinae and Bryocorinae. Arq. Mus. Nac..

[56] Carvalho J.C.M., Froeschner R.C. (1987). Taxonomic names proposed in the insect order Heteroptera by Jose Candido de Melo Carvalho from 1943 to January 1985, with type depositories. J. N. Y. Entomol. Soc..

[57] Schuh R.T. (1995). Plant Bugs of the World (Insecta: Heteroptera: Miridae).

[58] Schuh R.T. 2002–2013 Online Systematic Catalog of Plant Bugs (Insecta: Heteroptera: Miridae). http://research.amnh.org/pbi/catalog/.

[59] Carvalho J.C.M., Froeschner R.C. (1994). Taxonomic names proposed in the insect order Heteroptera by José Candido de Melo Carvalho from January 1989 to January 1993. J. N. Y. Entomol. Soc..

[60] Distant W.L. (1883). Insecta. Rhynchota. Hemiptera-Heteroptera. Biologia Centrali Americana.

[61] Carvalho J.C.M. (1959). Catalogue of the Miridae of the World. Part IV. Arq. Mus. Nac..

[62] Carvalho J.C.M., Dolling W.R. (1976). Neotropical Miridae, CCV: Type designations of species described in the “Biologia Centrali Americana” (Hemiptera). Rev. Bras. Biol..

[63] Carvalho J.C.M. (1989). Mirideos neotropicais, CCC: Generos e espécies novos da America Central e America do Sul (Hemiptera). Rev. Bras. Biol..

[64] Chérot F., Pauwels O.S.G. (2000). Les specimens-types de Miridae (Insecta: Heteroptera) des collections du Musee Royal de l’Afrique Centrale (Tervuren, Belgique). Doc. Zool..

[65] Carvalho J.C.M. (1989). Mirideos neotropicais, CCCIV: Novos generos e espécies do Brasil (Hemiptera). Rev. Bras. Biol..

[66] Wolski A., Henry T.J. (2015). Review and a new subfamily placement of the plant bug genus *Isometocoris* Carvalho and Sailer, 1954 (Hemiptera: Heteroptera: Miridae), with the description of a new species from Brazil. Proc. Entomol. Soc. Wash..

